# Plane Waves Versus Correlation-Consistent Basis Sets: A Comparison of MP2 Non-Covalent Interaction Energies in the Complete Basis Set Limit

**DOI:** 10.1021/acs.jctc.3c00952

**Published:** 2023-12-04

**Authors:** Justin Villard, Martin P. Bircher, Ursula Rothlisberger

**Affiliations:** † Laboratory of Computational Chemistry and Biochemistry, Institute of Chemical Sciences and Engineering, École Polytechnique Fédérale de Lausanne (EPFL), CH-1015 Lausanne, Switzerland; ‡ Computational and Soft Matter Physics, Universität Wien, A-1090 Wien, Austria

## Abstract

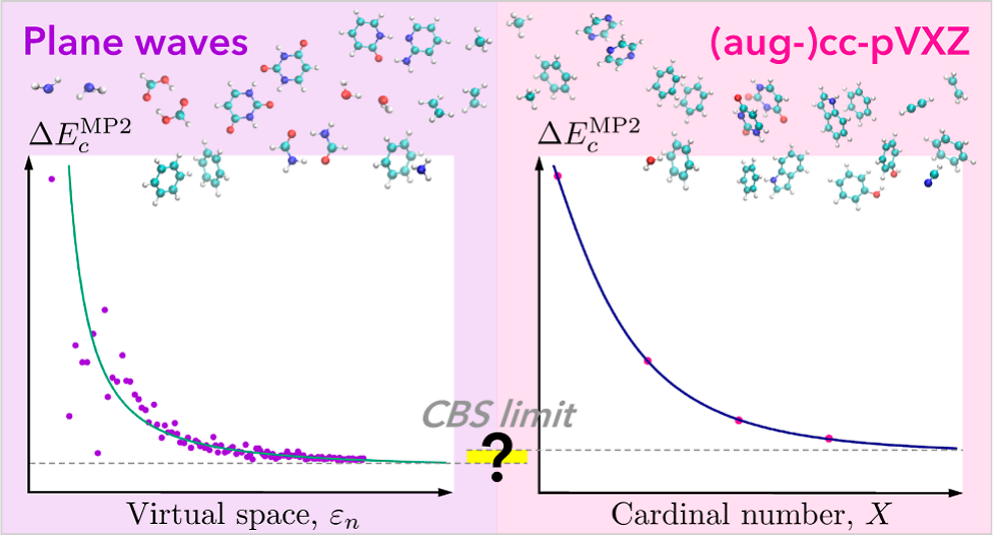

Second-order Møller–Plesset perturbation theory (MP2) is the most expedient wave function-based method for considering electron correlation in quantum chemical calculations and, as such, provides a cost-effective framework to assess the effects of basis sets on correlation energies, for which the complete basis set (CBS) limit can commonly only be obtained via extrapolation techniques. Software packages providing MP2 energies are commonly based on atom-centered bases with innate issues related to possible basis set superposition errors (BSSE), especially in the case of weakly bonded systems. Here, we present noncovalent interaction energies in the CBS limit, free of BSSE, for 20 dimer systems of the S22 data set obtained via a highly parallelized MP2 implementation in the plane-wave pseudopotential molecular dynamics package CPMD. The specificities related to plane waves for accurate and efficient calculations of gas-phase energies are discussed, and results are compared to the localized (aug-)cc-pV[D,T,Q,5]Z correlation-consistent bases as well as their extrapolated CBS estimates. We find that the BSSE-corrected aug-cc-pV5Z basis can provide MP2 energies highly consistent with the CBS plane wave values with a minimum mean absolute deviation of ∼0.05 kcal/mol without the application of any extrapolation scheme. In addition, we tested the performance of 13 different extrapolation schemes and found that the *X*^–3^ expression applied to the (aug-)cc-pVXZ bases provides the smallest deviations against CBS plane wave values if the extrapolation sequence is composed of points *D* and *T*, while  performs slightly better for TQ and Q5 extrapolations. Also, we propose  as a reliable alternative to extrapolate total energies from the DTQ, TQ5, or DTQ5 data points. In spite of the general good agreement between the values obtained from the two types of basis sets, it is noticed that differences between plane waves and (aug-)cc-pVXZ basis sets, extrapolated or not, tend to increase with the number of electrons, thus raising the question of whether these discrepancies could indeed limit the attainable accuracy for localized bases in the limit of large systems.

## 1 Introduction

Basis functions used in any ab initio calculation, whether they concern solids or molecules, are the algebraic pillars of the electronic wave function whenever the Schrödinger equation has to be solved numerically. Gaussian-type orbitals (GTOs) are by far the most popular basis functions in quantum chemistry due to their atomically localized analytical forms that allow for an efficient evaluation of the multielectron integrals appearing in wave function-based methods^1,2^ and, to a lesser extent, in Kohn–Sham density functional theory (DFT).^3,4^

Despite their numerical advantages, GTO basis sets are inherently nonorthogonal and prone to linear dependencies that become more pronounced as the size of the basis increases. In addition, the calculation of relative energies with atom-centered functions suffers from the basis set superposition error (BSSE)^1^ because of the completeness mismatch between systems of different sizes, which tends to overstabilize bound clusters relative to single fragments, thus overestimating binding energies. Schemes for estimating the BSSE and correcting for the basis set imbalance are therefore commonly employed, with the most standard approximation being the counterpoise (CP) correction.^5,6^ Nevertheless, it has been argued that such an ad hoc rectification can lead to spurious effects on the final accuracy,^6−14^ for instance, due to an unequal amount of correction between the entire system and its constituents.^14^ As a result, the CP correction applied to the calculation of interaction energies is sometimes viewed more as an estimate. Such an estimate can be considered neither an upper nor a lower bound for the actual BSSE.^8,11,15^

An additional complication with GTO bases is that their intrinsic construction is not necessarily systematic as their size increases, which possibly leads to difficulties in converging properties in a smooth and monotonic way, which is a known problem for Hartree–Fock (HF) or correlated methods such as, e.g., second-order Møller–Plesset perturbation (MP2)^16^ or coupled cluster (CC)^17^ energies. For this reason, Dunning’s cc-pVXZ correlation-consistent polarized bases^18^ (where *X* = D,T,Q,5,6 is the cardinal number), as well as their augmented aug-cc-pVXZ analogues containing diffuse functions,^19,20^ are among the most popular for post-HF approaches because of their meticulous design, which makes it possible to gradually recover a maximum of electron correlation by increasing the basis’ cardinality.

The development of such basis sets subsequently led to the proposal of numerous and mostly empirical extrapolation schemes to estimate values in the complete basis set (CBS) limit,^21−23^ some of which are detailed later in Section 2.3.1. Although the BSSE vanishes in the CBS limit, these extrapolation procedures are not intended to directly correct for it, but rather attempt to eliminate the error due to the finite basis that we call here the basis set incompleteness error (BSIE).^6,14,24^ While most extrapolation schemes are of a fully empirical nature, some are based on theoretical motivations about the leading behavior of approximate atomic wave functions, so their applicability to molecular systems requires that the correlation energy be dominated by the electron–electron (Coulomb) cusp and that assumptions are transferable to polyatomic systems.^1,22,25^ In addition, it is generally assumed that extrapolations are applicable from one correlated method to another. For example, the usual *X*^–3^ scheme introduced by Helgaker^1,26^ relies on the finding that the principal expansion of the helium configuration-interaction (CI) energy holds for energies obtained with correlation-consistent bases because both converge according to the principal quantum number *n* for this two-electron atom. Transferred to molecules, this one would then assume that lower-order terms as well as chemical bonding effects are negligible and that the expression applies equally to all system sizes and quantum chemical methods.^1^

As an alternative to cope with the BSIE, explicitly correlated methods^1,22,27,28^ (e.g., MP2-R12/F12) are particularly suited for fast convergence of correlation energies due to their correlating many-electron basis functions that depend explicitly on the interelectronic coordinates *r*_12_. R12 or F12 refers to whether the explicit two-electron functions (geminals) are given by linear or Gaussian-type expressions, respectively. Apart from the wave function Ansatz, the R12/F12 methods are similar to their standard counterparts and thus converge in theory to values very close to the CBS limit. However, such calculations are neither free of BSSE for smaller bases nor of linear dependencies for larger molecules, and they can suffer from numerical instabilities.^15,28^ In addition, while recovering most of the correlation energy for a much smaller number of basis functions, they may still have difficulty incorporating the very last bits required to reach very high accuracy (≤0.1 kcal/mol) due to the choice of the geminal and integral approximations, such as the resolution of identity or neglect of terms, that are necessary to maintain reasonable calculation costs.^1,22,26,28,29^ For instance, a deviation of about 0.5 kcal/mol was observed between H_2_O total energies coming from different MP2-R12 approximations, and a 1 kcal/mol difference was identified between different R12 basis sets.^26^ Deviations of the order of 0.1 kcal/mol were also noticed when it came to interaction energies.^28^ Nevertheless, MP2(CCSD(T))-R12/F12 calculations have so far been the only CBS references available to assess the reliability of GTO extrapolations from the (aug-)cc-pVXZ bases.^21,28,30^

In this work, we follow a different route to obtain converged values in the CBS limit by evaluating the MP2 correlation energy in a converged plane wave (PW) basis set. PWs have the advantage of forming an orthogonal basis, the completeness of which is established regularly and monotonically with the increase in a single parameter, the kinetic energy cutoff, regardless of the level of theory employed. Since the basis functions are fixed in space rather than being located at atomic centers, there is no BSSE from the outset, and the BSIE is systematically and progressively reduced to reach the ultimate intrinsic level of precision achievable by the quantum chemical method itself. In contrast, PWs generally describe only the valence electrons explicitly in order to reduce the number of basis functions and limit calculation costs, with pseudopotentials replacing the effects of the core electrons in accommodating the variations of the wave function near the nuclei that would require the inclusion of rapidly varying basis functions, i.e., high energy cutoffs leading to computationally unfeasible basis set sizes. Even if pseudopotentials are employed, a PW calculation can necessitate a number of basis functions of up to a few hundred times that of GTOs (typically of the order of 10^5^ PWs) for a similar level of convergence with respect to the basis set limit, thus requiring a highly optimized parallel implementation.^31,32^

In principle, whether obtained with GTOs or PWs, CBS-converged energies must be identical. However, fundamental differences exist between those two types of basis functions and have never been thoroughly compared. We hence attempt to fill this gap with the present work. More specifically, due to the CP correction and extrapolation schemes required for GTOs or due to some peculiarities of PWs in the treatment of isolated systems (where the interaction between periodic replicas intrinsic to PWs has to be explicitly removed), it is fundamental to clarify how the results obtained with these two different approaches may differ in practice. To answer this question, noncovalent interaction energies of dimer systems provide a sensitive test case because the description of weak dispersion interactions often requires an accuracy of the order of 0.05–0.5 kcal/mol.^1,33,34^ Such systems thus challenge the ability of a basis set to best capture the short-range components of the correlation energy around the Coulomb cusp while at the same time incorporating the long-range features of the intermolecular interactions. A priori, the delocalized and balanced nature of PWs seems more appropriate for such a treatment of, e.g., hydrogen-bonded and van der Waals complexes, but at the cost of a much larger number of basis functions. On the other hand, polarization and delocalization of the electronic wave function necessitate larger and/or augmented GTO bases for better coverage of real space.^22^ If these effects are accounted for, the presence of diffuse functions causes the basis set to be more prone to the BSSE, which also makes noncovalent interactions a problem of choice for examining the effect of the CP correction.

In the following, we first give some general information on how to obtain MP2 interaction energies in the CBS limit with PWs (Section 2.2) as well as with correlation-consistent GTOs (Section 2.3); thereafter, specific computational details are reported (Section 3). We then demonstrate how to efficiently and accurately converge MP2 relative energies in PW basis sets as implemented in the CPMD software^35^ (Section 4.1) and present the results of applying this approach to the calculation of noncovalent interaction energies of 20 systems from the S22 benchmark set,^33,34^ which we then compare to their (aug-)cc-pVXZ analogues of different sizes *X* = D,T,Q,5 (Section 4.2). In the following, we will call our test subset S22* for brevity’s sake. We then searched among 13 GTO extrapolation schemes reported in the literature in order to identify those that agree the best with PWs in the CBS limit (Section 4.3) and investigate the capability of new, different extrapolation laws (Section 4.4). We find that the CBS limits reached with the best GTO extrapolations and PWs show no significant difference, i.e., they do not deviate by more than 0.2 kcal/mol for all systems studied herein. However, it is observed that some residual deviations increase with system size (Section 4.5). Finally, we conclude with some general recommendations concerning the choice of correlation-consistent basis sets for the calculation of correlated energies in the CBS limit (Section 5).

## 2 Methods

### 2.1 Second-Order Møller–Plesset Perturbation Theory

In Møller–Plesset perturbation theory, the dynamic correlation energy is estimated as a series of perturbative terms originating from Rayleigh–Schrödinger perturbation theory around a zero-order Hamiltonian given by the sum of Fock operators.^16^ By taking the ground state Slater determinant that solves the HF problem as the unperturbed wave function, the total electronic energy *E* is approximated at second order by the sum of the HF energy and the second-order Møller–Plesset (MP2c) correlation contribution
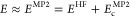
1

As a consequence of Brillouin’s theorem, single excitations of the HF reference do not couple to the HF ground state determinant, and only doubly excited determinants contribute to the MP2 correlation (MP2c) energy, leading to an expression that includes double sums over occupied and virtual molecular orbitals. For the spin-restricted case, where two opposite-spin electrons occupy the same spatial orbital, the expression reads^2,36^
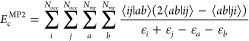
2where *i* and *j* denote (valence-only) spatially occupied orbitals ϕ_*i*,*j*_, and *a* and *b* denote their virtual counterparts that are all eigenstates of the Fock operator with respective eigenvalues ε_*i*,*j*,*a*,*b*_. The numerator in eq 2 accounts for Coulomb-type interactions between occupied-virtual pairs of orbitals in the evaluation of two-electron integrals
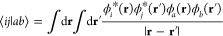
3

The evaluation of this term in atom-centered basis sets is straightforward and documented elsewhere.^1,2^

### 2.2 PW Basis Set

In this work, we address the calculation of the MP2 energy for isolated systems in the PW basis set, considering the Γ-point sampling of the Brillouin zone only; orbitals are therefore expanded in reciprocal space as
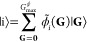
4with the reciprocal space (**G**) coefficients  that are the Fourier components of the molecular orbitals and a PW ⟨**r**|**G**⟩ = Ω^–1/2^ e^i**G**·**r**^ in the simulation supercell of volume Ω. Computationally, the infinite basis is truncated by restricting the maximum norm *G*_max_^ϕ^ of **G** vectors to respect
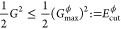
5

The wave function cutoff energy *E*_cut_^ϕ^, as well as the volume Ω (both user-defined and system-dependent), act as parameters to ensure the convergence of the energy with respect to the basis size. The number *N*_**G**_ of basis functions is dictated by the following estimate^37^
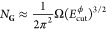
6

In reciprocal space, the two-electron integrals (eq 3) can be evaluated with linear scaling with respect to *N*_**G**_ since the Coulomb operator takes the diagonal form
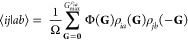
7where Φ(**G**) is the generalized Coulomb potential that depends on the dimensionality and boundary conditions of the system studied, which we describe in more detail in Section 2.2.2. The overlap pair densities appearing in eq 7 are obtained from the Fourier transforms
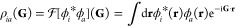
8which are replaced by fast Fourier transforms (FFTs) in the case of the discrete representation of the ϕ_*i*_. In principle, because the charge density ρ depends on the square of the orbitals, the maximum radius for the density expansion in the reciprocal space should be as high as 2*G*_max_^ϕ^, meaning that a density cutoff energy *E*_cut_^ρ^ = 4*E*_cut_^ϕ^ (cf. eq 5) is needed to maintain a consistent resolution between orbitals and densities in reciprocal space.^37^ For example, a factor of 4 is used here by default in the calculation of the exchange and Coulomb integrals involved in the zero-order HF calculation. For the MP2 correlation energy (eq 2), whatever the ratio between  and *E*_cut_^ϕ^, the canonical scaling in the PW basis set remains quintic and behaves like  if the pair densities ρ_*ia*_(**G**) are precalculated and stored in memory. Consequently, the number  of **G** vectors entering the expansion of ρ_*ia*_ has a drastic effect on the overall performance of the MP2 energy evaluation, as it also affects the prefactor and memory requirements. For this reason, it is imperative to study below to what extent a reduction in  alters the accuracy of the MP2 energy (Section 4.1).

#### 2.2.1 Extrapolation of PWs to the Basis Set Limit

The convergence of post-HF energies with respect to the number of GTO basis functions is markedly slower than it is for HF or DFT. This is attributed to the need for large atom-centered bases to fully accommodate the asymptotic behavior of the wave function around the electron–electron cusp.^1^ For PWs associated with effective pseudopotentials, we observe that the value of *E*_cut_^ϕ^ that converges relative HF energies is in general close to that required for recovering most of the MP2c contribution, and both require a fairly large *N*_**G**_. In contrast to atom-centered bases, the sluggish convergence of the MP2 energy in PWs is rather reflected in the progression of the sum
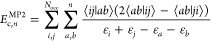
9with respect to the contribution of an additional virtual orbital *n* (≤*N*_vir_). In theory, the effective Hilbert space defined by the numerical basis has the size *N*_**G**_ = *N*_occ_ + *N*_vir_, and the virtual space is the algebraic consequence of the basis set being larger than the number of electrons in the system. As an illustration, the MP2 calculation with the largest basis set considered in this work has *N*_**G**_ = 408 126 and *N*_occ_ = 37, which makes the entire Fock matrix diagonalization and the direct evaluation of eq 2 simply intractable (*N*_vir_ = 408 089, ∼10^13^ summands, ∼6 × 10^12^ρ_*ia*_(**G**) points to be stored with double precision in 45 TB of RAM). For comparison, the same calculation with the GTO aug-cc-pV5Z basis set requires only 2945 basis functions. Therefore, the enormous size of the basis set coupled with the steep scaling of the methods constitute the main challenges when carrying out correlated calculations with PWs.

Fortunately, as it was established by numerical^38−42^ and analytical^43^ considerations, the PW correlation energy can be extrapolated to the CBS limit with respect to the virtual orbitals. Relying on the model of the homogeneous electron gas (HEG) in a finite cell, Shepherd et al. showed that the MP2 correlation energy in the large basis set limit (*E*_cut_^ϕ^ → ∞) behaves like^43^
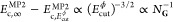
10

By noticing that the eigenstates of the HEG Fock matrix are nothing else than pure PWs |**G**⟩, any HF orbital of a many-electron system can be interpreted as the results of a unitary transformation of the HEG HF problem, so that the same extrapolation law generalizes to single-reference quantum chemical methods of solids and molecules (in the limit of a complete and sufficiently large basis).^43^ In another interpretation, one can assume that the virtual states of very high (continuum) energy lose their molecular character and become closer to PWs, so that their contributions to the correlation energy resemble those of the HEG. Based on that, the same authors proposed a single-point extrapolation of eq 9, from intermediate points of a single calculation, that converges smoothly and reliably to the basis set limit according to
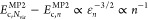
11

At *E*_cut_^ϕ^ (*N*_**G**_) sufficiently large, *N*_vir_ is large enough to recover the CBS energy, and ε_*n*_ acts as the cutoff energy of an auxiliary basis which is gradually expanding toward the CBS limit. The fact that the orbitals and eigenvalues of eq 9 originate from the complete basis has no effect on extrapolation in practice. This technique has the advantage of considerably truncating the virtual space required to calculate the MP2 correlation energy since a maximum *n* of the order of *n*_max_ = 10 000–20 000 is satisfactory for extrapolating relative energies. In addition, the Fock operator must be diagonalized only for the *n*_max_ orbitals with the lowest eigenvalues.^44−46^

Despite this, MP2 computations still involve the storage of  values of the pair densities (eq 8) for calculating the integrals over  ∼10^4^ to 10^5^ integrands (eq 7) as well as the contribution of ∼10^9^ to 10^11^*ijab* sums (eq 9) so that only a parallel approach can handle such intense RAM and CPU requirements in a reasonable time. The PW/pseudopotential MP2 method used herein has been developed and implemented in the CPMD program,^35,47^ for which we give the pseudocode of the parallel implementation in Supporting Information (Algorithm 1).

#### 2.2.2 Treatment of the Coulomb Potential for Isolated Systems

The PW basis set offers the possibility to evaluate the nonlocal and cumbersome Coulomb potential  in reciprocal space, with its Fourier transform (sampling the Γ-point only) being^37^
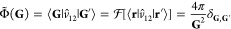
12but the long-range nature of the Coulomb interactions in direct space poses problems for the evaluation of multicenter integrals such as those appearing in HF^48−53^ or MP2 correlation energy.^39^ Indeed, discrete sums of the type of eq 7 with Φ(**G**) = Φ̃(**G**) are facing a singularity in **G** = 0, which is only properly integrable in the thermodynamic limit (Ω → ∞, ∑_**G**_ → Ω/(2π)^3^∫d**G**) and is a consequence of the finite simulation cell imposed by the numerical computation. Simply ignoring the problematic component makes the convergence of the integrals very slow and requires either many replicas of the unit cell (large supercell) or a much finer and more careful sampling of the eventual **k**-point mesh. Therefore, schemes have been suggested in order to screen the **G** = 0 divergence and obtain faster convergence.^48,49,52^

In the context of hybrid functionals, be it for isolated or periodic systems, Broqvist et al. (BAP)^53^ proposed to use an auxiliary function *f*(**G**), which acts as a singularity correction by transforming the summand of eq 7 regular function with
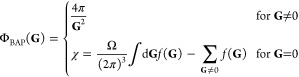
13and the function chosen as
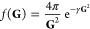
14such that most of the singularity is retrieved for *f*(**G**) → Φ̃(**G**), meaning that
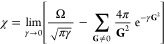
15allows faster convergence of the integrals with respect to the supercell volume. For isolated systems, the symmetry of the “fictitious” supercell and its volume Ω are therefore the adjustable parameters to converge the exchange-like integrals with the aim of removing the electrostatic interactions between periodic images when the box size increases. In that case, it has been shown that the correction χ of the singularity in the Coulomb potential greatly improves the convergence of the total energy as well as the HOMO–LUMO gap with respect to the supercell size, as opposed to simply neglecting the **G** = 0 component. Hence, we focus on the behavior of this correction when applied to the MP2 correlation energy.

An alternative treatment specific to isolated molecules is the effective decoupling of the Coulomb interactions between the system and its unphysical periodic replicas. For this purpose, special Poisson solvers^54−57^ provide an expression of the potential induced by the cluster charge density when modeled in an infinitely replicated periodic setup. The method of Martyna and Tuckerman (MT)^56^ assumes that the density vanishes far enough from the boundaries of the box so that the potential can be seen as having the same periodicity as the simulation domain *D*. In this first/nearest image picture, the potential in the MT method converges toward the isolated system limit when the supercell is sufficiently expanded. Separating its action at short and long distances with the help of the parameter α (1/*r* = [erfc(α*r*) + erf(α*r*)]/*r*), the latter can be recast as
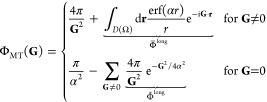
16where the new terms that add up to  come from the difference between the Fourier transform  and the Fourier series components  of the long distance part, which acts as a screen of the interactions between the isolated system and its infinite periodic images. Note that the singularity of Φ̃(0) would be exactly canceled by that of , and both were ignored in eq 16, but the nonsingular difference  must be included. It has been shown in practice that for α*L* ∼ 7, where *L* is the smallest size of the parallelepiped box,  can be efficiently evaluated via a FFT that converges rapidly with respect to the Cartesian grid. In the framework of PW/pseudopotential DFT, the evaluation of Coulomb-like integrals with the MT Poisson solver provides accurate energies, provided that the integration domain spans about twice the size of the electron density. Thus, as noted by MT, increasing the size of the supercell becomes analogous to converging the energy according to the largest-width diffuse function included in a Gaussian basis set.

BAP (eq 13) and MT (eq 16) schemes look very similar for **G** = 0, where the sum over **G** ≠ 0 vectors actually corresponds to the electrostatic energy of a Gaussian charge distribution interacting with a compensating uniform background in a periodic setup.^53^ The first term in eq 15 accounts for the electrostatic self-energy of an isolated Gaussian charge in the supercell, so intuitively, χ corrects the singularity using the difference between the electrostatic energy of an isolated probe charge and its periodically repeated analogues in a compensating background.^53,58^ For MT, instead, the Gaussian charge distribution is used to construct the screening function  of the long-range electrostatic interactions after an Ewald-type splitting of the Coulomb potential.^59,60^ Very few analyses of the BAP or the MT treatment have been performed on HF calculations with full exact exchange, and to the best of our knowledge, none has been done on the MP2 correlation energy. It is therefore crucial to understand how these act on such energy contributions to ensure the convergence and accuracy of PW results in what follows.

### 2.3 Correlation-Consistent GTO Basis Sets

In the realm of GTOs, the cc-pVXZ^18,20^ and aug-cc-pVXZ^19,20^ basis families of Dunning and co-workers have been designed to recover most of the correlation energy due to the valence electrons. More precisely, these are called correlation-consistent since the basis functions that are added at each level of cardinality *X* = D, T, Q,... contribute similar amounts of energy, independently of their type (*s*, *p*, *d*, ...), and in a consistent manner even in the presence of polarization or diffuse functions. All of these are optimized so as to maximize their contributions to the atomic correlation energy. For example, to balance the set, *s* and *p* functions are added when the polarization space is extended so that the correlation energy error due to the *s* and *p* functions does not exceed the error from the polarization space. The exponents of such *s* and *p* functions are optimized with respect to atomic HF energies, while the correlating polarization functions come from valence energy minimization at the atomic CISD level of theory. The aug-cc-pVXZ bases are derived from the original cc-pVXZ, with the addition of one diffuse function per angular momentum present in the set, whose (smaller) exponents are determined by minimizing the atomic CISD energy of anions. Although first intended for a better description of electron affinities, the aug-cc-pVXZ basis family has been shown to progressively enhance the convergence of other molecular properties such as proton affinities,^61^ dipoles, and polarizabilities,^62−64^ or energies of weakly bound systems.^11,63,65−67^

The main advantage of the (aug-)cc-pVXZ basis sets is their ability to converge results toward the basis set limit in a (semi)systematic manner at the cost of increasing the number of contracted basis functions, *N*_b_. For first-row atoms, this latter increases with the cardinal number *X* as^26^
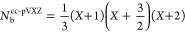
17
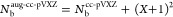
18such that, for a computer time associated with MP2 that scales as *N*^5^*N*_b_^4^, where *N* is the number of atoms, improving the correlation energy with a larger basis grows as *N*^5^*X*^12^. Q, 5, or 6 zeta calculations may therefore be prohibitively expensive for larger systems of interest.^68−70^

This is in contrast to PWs, for which the basis size does not depend explicitly on the number of atoms *N* but only on the volume of the supercell and the cutoff energy so that the MP2 energy scales as  (Section 2.2). Assuming that a sufficiently high energy cutoff may be chosen to faithfully describe pair densities over a wide range of systems and that *n*_max_ increases less than linearly with *N* (as we have observed^47^), the PW basis set then becomes more favorable in the limit of large systems,^2^ provided that Ω does not increase significantly for the correct convergence of Coulomb interactions in a periodic setup (Section 2.2.2).

#### 2.3.1 Extrapolations of GTOs to the Basis Set Limit

Although the correlation-consistent basis sets provide gradual and monotonic progress, their associated computational cost grows faster than the rate of convergence. As a rule of thumb,^22^ it is globally said that an improvement of the energy accuracy by a factor of 10 necessitates a computational effort increased by a factor of 10^4^, and the convergence of the correlation energy, be it MP2, CCSD, CCSD(T), ..., remains so slow that basis set limit estimates can only be reached by extrapolation.^21−23,71^ As a consequence, expressions based on the comparison with very large basis calculations or, most commonly, with explicitly correlated methods (e.g., MP2-R12/F12 and CCSD(T)-R12/F12) have been suggested, but the computational overhead for obtaining such accurate references has often restricted the size of the validation systems to a few dozen electrons.

**Scheme 1:**
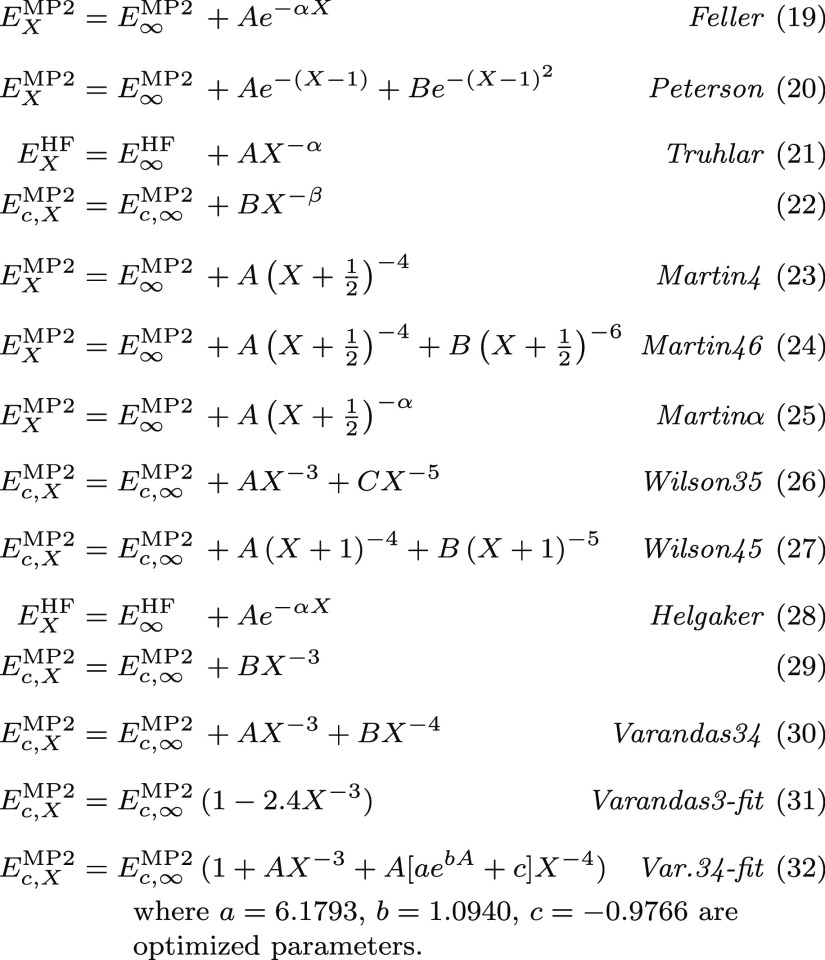
Extrapolation Expressions Tested in This Work for the (aug-)cc-pVXZ Basis Sets

Even though nonexhaustive, we report in Scheme 1 some formulas found in the literature to estimate correlated energies in the basis set limit when extrapolated according to *X* = 2(D), 3(T), 4(Q), 5, ... First proposals by Feller et al. (eq 19),^11,21,72−74^ Peterson et al. (eq 20),^64,75−77^ and Truhlar (eqs 21 and 22)^69^ are all based on empirical interpolations of the total energy, with the difference that Truhlar suggested different powers for the convergence of the HF and MP2c energies. All expressions contain three parameters and, therefore, require at least three points for extrapolation. However, in the case of the expressions from Truhlar, α = 3.4 and β = 2.2 were found to provide a minimal RMSD with respect to MP2-R12 energies of small systems so that these can also be used for two-point extrapolations (e.g., DT, TQ, Q5). In some cases, it has been argued that the CBS values calculated directly from relative rather than total energies are more accurate,^21,64^ but no clear explanation or justification was provided to support that claim.^30^

On the other hand, one possible theoretical motivation holds its origin in the partial-wave expansion of a two-electron atom:^1,22,23^ By treating the Hamiltonian of the bare nucleus of the helium atom as zero-order and the electron interaction as a perturbation, Schwartz studied the convergence of the correlated atomic wave function on a one-electron basis.^22,78^ He established that the partial wave increments δ*E*_*l*_^(2)^ to the second-order energy  follow an asymptotic formula in the limit of large *l*, which behaves like
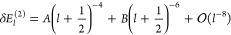
33with *l* being the degree of Legendre polynomials entering the partial wave expansion of the first-order wave function. Interestingly, the *l*-th component in the partial wave expansion corresponds to a one-electron atomic function with angular momentum *l*.^79^ Translated to many-electron atoms, this implies that δ*E*_*l*_^(2)^ is equivalent to the energy increase due to the addition of a saturated shell of basis functions of angular momentum *l* to the basis set that expands the first-order wave function. For standard electronic structure methods (e.g., MPn^79^ or CI^80,81^) and many-electron atoms, similar forms were derived with odd terms that may also arise, where one makes the general assumption that the increment of the correlation energy can be expanded as
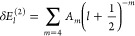
34with numerical coefficients, *A*_*m*_. In the limit of large *L*, with the omission of all basis functions with *l* > *L*, the error on the correlation energy resulting from the basis set truncation can therefore be estimated as^22,70^
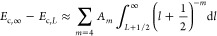
35
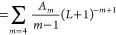
36which consequently describes the asymptotic limit of the energy for consecutive enlargements of the basis set. This stands under the assumption that each increment of the basis set contains all functions covering the atomic angular momentum up to *L*. However, choosing atomic angular momentum as the parameter for assessing energy convergence is questionable when generalizing it to molecules. Moreover, this quantum number is not consistent with the construction of the (aug-)cc-pVXZ bases, which rather involves successive increments of functions with different angular momenta (Section 2.3).

In spite of this, expressions inspired by eq 36 have demonstrated their potential for the extrapolation of (aug-)cc-pVXZ energies to the basis set limit, also in the case of polyatomic systems. For example, Martin et al. proposed to average between hydrogen, helium (*L* ∼ *X* – 1), and first-row (*L* ∼ *X*) atoms to replace *L* by , yielding eqs 23 and 24 (Scheme 1),^25,82^ which correspond to the leading orders found by Kutzelnigg et al. for the MP2 energies.^79^ He later suggested that the quality of the results can improve if the HF and MP2c energies are processed separately with eq Martinα (25).^83^

Furthermore, by comparing the MP2-R12 references of Ne, HF, H_2_O, and N_2_, Wilson and Dunning found that the ansatz
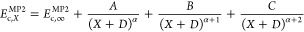
37was giving the best match for (α = 3, *B* = 0, and *D* = 0) and (α = 4, *C* = 0, and *D* = 1), consequently proposing Wilson35 (26) and Wilson45 (27) for extrapolating correlated energies.^30^

Alternatively, Helgaker et al. put forward the use of eq Helgaker (29),^15,26,29,84^ which corresponds to the leading order of eq 36, and the identification of *L* ∼ *X* – 1 based on a better agreement with R12 results.^26^ This allows the extrapolation of the correlation energy from a two-point linear fit and was later motivated by analyzing the energy increments of the principal expansion of the ground-state helium atom, yielding in this case a convergence with respect to the principal quantum number *n*, which is a priori more in line with the progressive construction of the (aug-)cc-pVXZ bases.^1,22^ Since the number of basis functions increases cubically with *X* (eq 17), Helgaker (29) is equivalent to a convergence of the correlation energy as a function of 1/*N*_b_. Interestingly, this is analogous to PWs, for which the correlation energy converges to 1/*N*_**G**_ (eq 10). A different rate of convergence was observed for the HF energy, so Helgaker argued for its separate treatment, according to Helgaker (28).^26,84^

Finally, Varandas investigated the universality of the *B* parameter in Helgaker (29) and found that eq Varandas34 (31) minimizes the difference with a set of CCSD(T)/MP2 energies of small molecules and different basis sets.^70^ However, the match was better if considering a fourth-order term with the general form of Varandas34 (30) or by exploiting an empirical interdependence between the parameters that leads to eq Var.34-fit (32), which has the advantage of requiring only two points for extrapolation.

Subsequently, we will examine which of these extrapolations provide better or worse agreement between the GTO and PW MP2 interaction energies in the CBS limit.

## 3 Computational Details

The geometries of the test systems were taken from the paper defining the S22 data set.^33^ Like in the original work on the S22 data set and its revised version,^34^ deformation energies are neglected, monomer structures are kept identical in the dimer configuration, and no monomer relaxation is carried out. To save computational resources, we opted to exclude the two adenine–thymine dimers from our test set, resulting in a set of 20 structures that we refer to as S22*.

### 3.1 PW Basis Set

A development version of CPMD 4.3 has been used for all MP2 calculations with PWs^35^ in combination with hard norm-conserving Goedecker–Teter–Hutter (GTH)^85^ pseudopotentials specifically parametrized for HF.^86^ The HF wave function has been optimized with either DIIS^87^ or preconditioned conjugate gradient optimization up to a maximum residual component of the gradient on occupied orbitals lower than 10^–7^ au, respectively, 10^–5^ au for the *n*_max_ virtual orbitals obtained via subsequent Davidson diagonalization.^46^

The MP2c contribution to the interaction energy of the *AB* complex is extrapolated according to eq 11, i.e., the extrapolation is performed on Δ*E*_c,*n*_^MP2^ = *E*_*AB*,c,*n*_^MP2^ – *E*_*A*,c,*n*_^MP2^ – *E*_*B*,c,*n*_^MP2^, which greatly accelerates the energy convergence compared to individual extrapolations. This allows us to set smaller *n*_max_ virtual orbitals to be diagonalized and processed in the MP2c double summation, which drastically reduces the computational requirements. No significant difference (>0.001 kcal/mol) was observed if the extrapolation was done as a function of *n*^–1^ or ε_*n*_^–3/2^, the latter eigenvalues corresponding to the dimer being therefore used. Extrapolation points are spaced by an increment of 100 virtual orbitals, and better accuracy is obtained with a linear fit according to
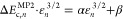
38where α = Δ*E*_c_^MP2^ recovers the PW CBS MP2c energy by ensuring that *n*_max_ is chosen large enough in order for eq 38, respectively eq 11, to be valid. For the systems studied, *n*_max_ is between 10 000 and 20 000. To account for the sensitivity of the extrapolated value with respect to the fitting range, the results from all possible intervals ending at *n*_max_ are calculated, and the final Δ*E*_c_^MP2^ value is averaged among the intervals that respect eq 38.

The cutoff energy *E*_cut_^ϕ^ of the wave function has been set to 150 Ry, and the density cutoff is set to the usual E_cut_^ρ^ = 4*E*_cut_^ϕ^ for all systems and supercell sizes. No change larger than ∼0.01 kcal/mol was observed on the extrapolated MP2 interaction energies at larger cutoffs (Table S1 in the Supporting Information). The effects of the cutoff energy for the MP2c pair densities, the supercell dimensions, and the decoupling between periodic images are discussed below in Section 4.1.

### 3.2 Correlation-Consistent GTO Basis Sets

The (aug-)cc-pVXZ calculations were performed with Orca 5.0.3,^88,89^ or for the larger systems and augmented bases, with Turbomole V7.1^90^ after checking that both programs gave identical results. The HF wave function and energies are obtained for all-electron calculations, while the frozen-core approximation is used for the MP2 correlation energy, i.e., occupied orbitals corresponding to core electrons are omitted in the MP2c evaluation. The convergence threshold for the SCF wave function was set to VeryTightSCF for Orca, respectively, and to 10^–7^ au for the energy gradient in Turbomole.

The CP correction scheme is used to correct for the BSSE.^5,6^ Therefore, uncorrected and BSSE-corrected interaction energies (HF or MP2c) are calculated as follows
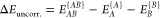
39
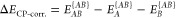
40
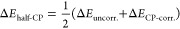
41where *E*_*A*_^{*A*}^ designates the energy of monomer *A* calculated in its basis {*A*}, and *E*_*A*_^{*AB*}^ its corrected energy calculated in the full {*AB*} basis that includes *ghost* functions located on system *B*. Since it was noticed that Δ*E*_uncorr._ and Δ*E*_CP-corr._ may converge to the basis set limit from opposite sides, it is sometimes assumed that the average Δ*E*_half-CP_ energy provides faster convergence,^15,91−93^ a strategy that we also examine.

## 4 Results and Discussion

### 4.1 Converging Accurate MP2 Energies with PWs

In this section, we discuss a number of technical details that are essential for making calculations of the MP2 interaction energies with PWs tractable. As already mentioned, the leading computational effort for this task scales as , for which *n*_max_ is reduced by the joint extrapolation of relative energies in the virtual space. Moreover, Ω and  define the number  of ρ_*ia*_(**G**) pair density values (eq 6) to be stored for the two-electron integrals (eq 7) and therefore have a strong influence on the memory requirements.

The effect of  on the correlation energy is reported in Table 1, which shows that no difference greater than 0.01 kcal/mol results from reducing  to the wave function cutoff energy *E*_cut_^ϕ^ (150 Ry) for various systems and supercell volumes. This amounts to projecting the pair densities, which require fewer high-frequency components, onto an auxiliary basis set for efficient computation of integrals, similar to what is done, for example, in the resolution of identity with GTO approaches (e.g., RI-MP2).^94^ We strongly emphasize the great benefit of such a reduction; in the case of, e.g., the parallel-displaced (PD) benzene dimer, computing the MP2 energies with  Ry is simply impossible on 25 nodes with 128 GB of memory each, while all test systems reported below could be evaluated with such a setup by fixing  to 150 Ry from now on.

**Table 1:** MP2c Contribution to the MP2 Interaction Energy for Selected Systems from the S22 Set and Pair Density Cutoff Energies a

S22 system	*r*_*x*_ *r*_*y*_ *r*_*z*_	Ω [Å^3^]	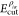 [Ry]	Δ*E*_c_^MP2^	σ_c_^MP2^
(NH_3_)_2_	2.0 2.0 2.0	987.84	150	–1.763	0.004
			300	–1.762	0.004
			600	–1.762	0.004
(H_2_O)_2_	1.7 1.9 2.2	648.86	150	–1.354	0.004
			300	–1.347	0.004
			600	–1.347	0.004
formic acid	1.5 1.6 2.3	953.50	150	–3.093	0.013
			300	–3.095	0.013
formamide	1.4 1.4 1.4	539.82	150	–3.658	0.004
			300	–3.655	0.005
			600	–3.655	0.006
PD benzene	1.8 1.8 1.8	2913.80	150	–10.470	0.021
			300	–10.461	0.024

a Energies are in kcal/mol, obtained with the MT Poisson solver. *r*_*x*,*y*,*z*_ are the respective *x*, *y*, *z* ratios of the orthorhombic supercell dimensions with respect to the HF electron density measured at an isosurface of 0.002 au, while Ω is the volume of the supercell. σ_c_^MP2^ corresponds to the standard deviation of Δ*E*_c_^MP2^ values extrapolated on different fitting ranges in the virtual space.

**Figure 1:**
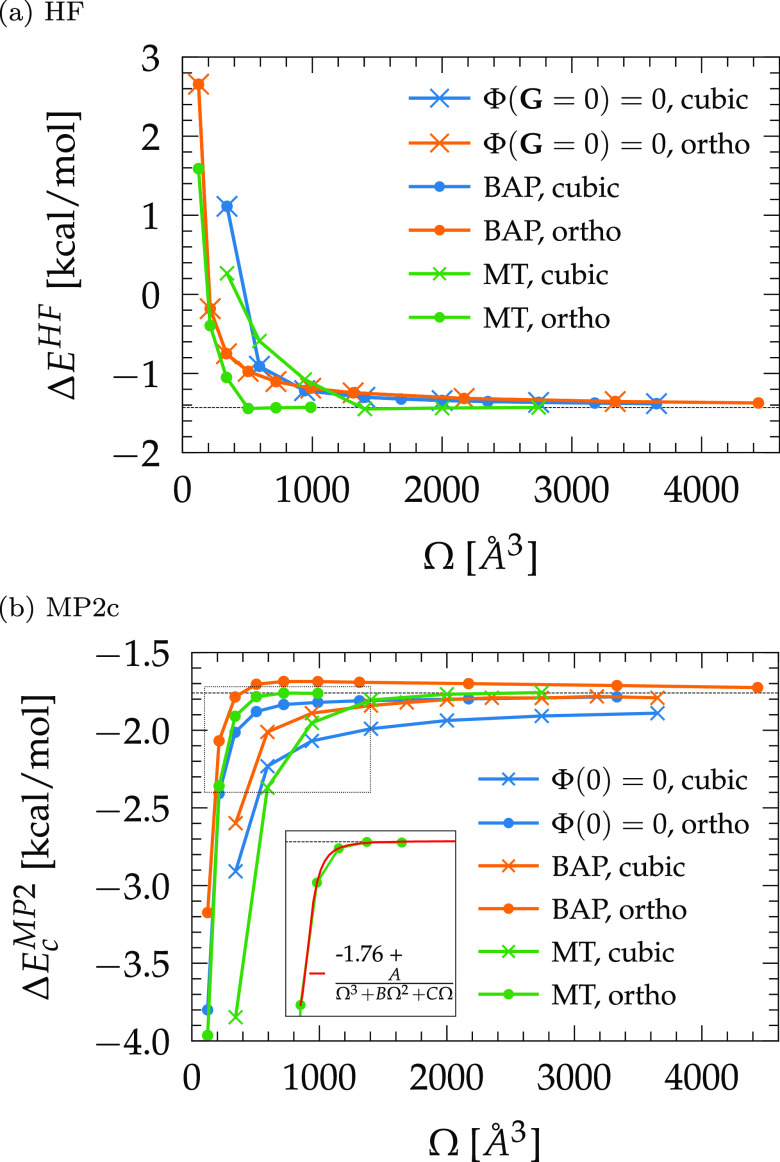
(a) HF and (b) MP2c contributions to the MP2 interaction energy of the NH_3_ dimer for different exchange (Coulomb) potentials. Ω is the volume of expanding cubic or orthorhombic (ortho) supercells around the dimer electron density.

The last parameter affecting the computational cost is the supercell volume, which should be as small as possible while accurately decoupling the interactions between periodic images of the system. As we saw, the choice of Ω is mainly dictated by the treatment of low-frequency components of the Coulomb operator acting in exchange-like integrals (Section 2.2.2). Figure 1 shows that significant differences exist between the BAP (eq 13) and MT (eq 16) potentials for converging interaction energies. As already observed,^53^ BAP greatly accelerates the convergence of the total HF energy compared to the simple neglect of the **G** = 0 component (Figure S1). However, both schemes perform identically when it comes to the HF interaction energies (Figure 1a). This is explained by computing the BAP correction
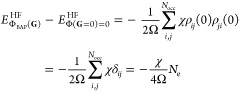
42that is proportional to the number of electrons *N*_e_ in the system and consequently cancels out between the energies of the dimer and monomers. Therefore, although beneficial for total energies, the BAP scheme does not improve the convergence of HF interaction energies for both cubic and orthorhombic boxes and necessitates large volumes to recover the last fraction of the mean field energy. Moreover, although a cubic box expansion with BAP accelerates the MP2c convergence against Φ(0) = 0 (Figure 1b), the BAP correction with an orthorhombic box makes it converge more slowly and nonmonotonically. This is a consequence of the BAP singularity correction (eq 15) that may switch sign depending on if the repulsion between the repeated Gaussian charge images or their attraction with the compensating background dominates according to the elongation of the cell.^53^ This demonstrates that the convergence behavior of the MP2 correlation energy for various supercell sizes and symmetries is nontrivial when resorting to effective Coulomb potentials.

In comparison, the MT potential performs better and is consistent between HF and MP2c contributions, most presumably thanks to the explicit cancellation of the **G** = 0 singularity and the directional effects of the screening function . Expanding an orthorhombic box around the electron density coupled to the MT Poisson solver allows to converge the MP2 interaction energy for a much smaller volume/computational cost, e.g., with a reduction factor of approximately 3 to 4 against other schemes for the NH_3_ dimer test system. In addition, it has been found that the MP2c energies of all systems considered herein can be extrapolated at large Ω according to
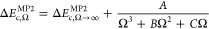
43as illustrated in the inset of Figure 1b. While it is well established that the MT potential requires the supercell to span at least twice the size of the density to converge DFT energies,^35,56^ our results show for the first time that the same criterion also applies to the MP2 energy. For practical information, all MP2 interaction energies considered in this work are converged to within 0.07 kcal/mol when setting the orthorhombic cell dimensions to *r*_*x*,*y*,*z*_ = 1.8 times the extent of the density, measured at an 0.002 au isosurface. For very high accuracy (∼0.01 kcal/mol), a ratio of 2.2 is recommended instead. Hence, eq 43 is of significant help to ensure the recovery of the last fraction of the correlation energy and becomes indispensable for the treatment of larger systems that would impose a too large box size and intractable computational cost.

Within these settings, we have shown how various factors can push the limits of MP2 calculations with PWs. The first factor consists of truncating the virtual space thanks to an analytical extrapolation (eq 11); the second relates to the reduced number of PWs necessary to expand the pair densities; and finally, the last refers to the choice of an efficient Coulomb operator for treating isolated systems and correlation energies. Thanks to these findings, it has been made possible to access the MP2 interaction energies of systems with up to ∼100 electrons that are listed in Table S2 of the Supporting Information. Convergence was achieved by progressively expanding an orthorhombic cell (*r*_*x*,*y*,*z*_ = 1.2, 1.4, 1.6, 1.8, 2.0, and 2.2 when possible) with the MT Poisson solver. The HF components were retained when no variations larger than 0.01 kcal/mol were measured, while MP2c contributions were extrapolated first via eq 38 and then via eq 43. Standard deviations due to this extrapolation procedure are also reported in Table S2 and do not exceed 0.05 kcal/mol for energies spanning a range of −0.50 to −20.19 kcal/mol.

### 4.2 HF/MP2 Energies in PWs versus GTO Bases

**Figure 2:**
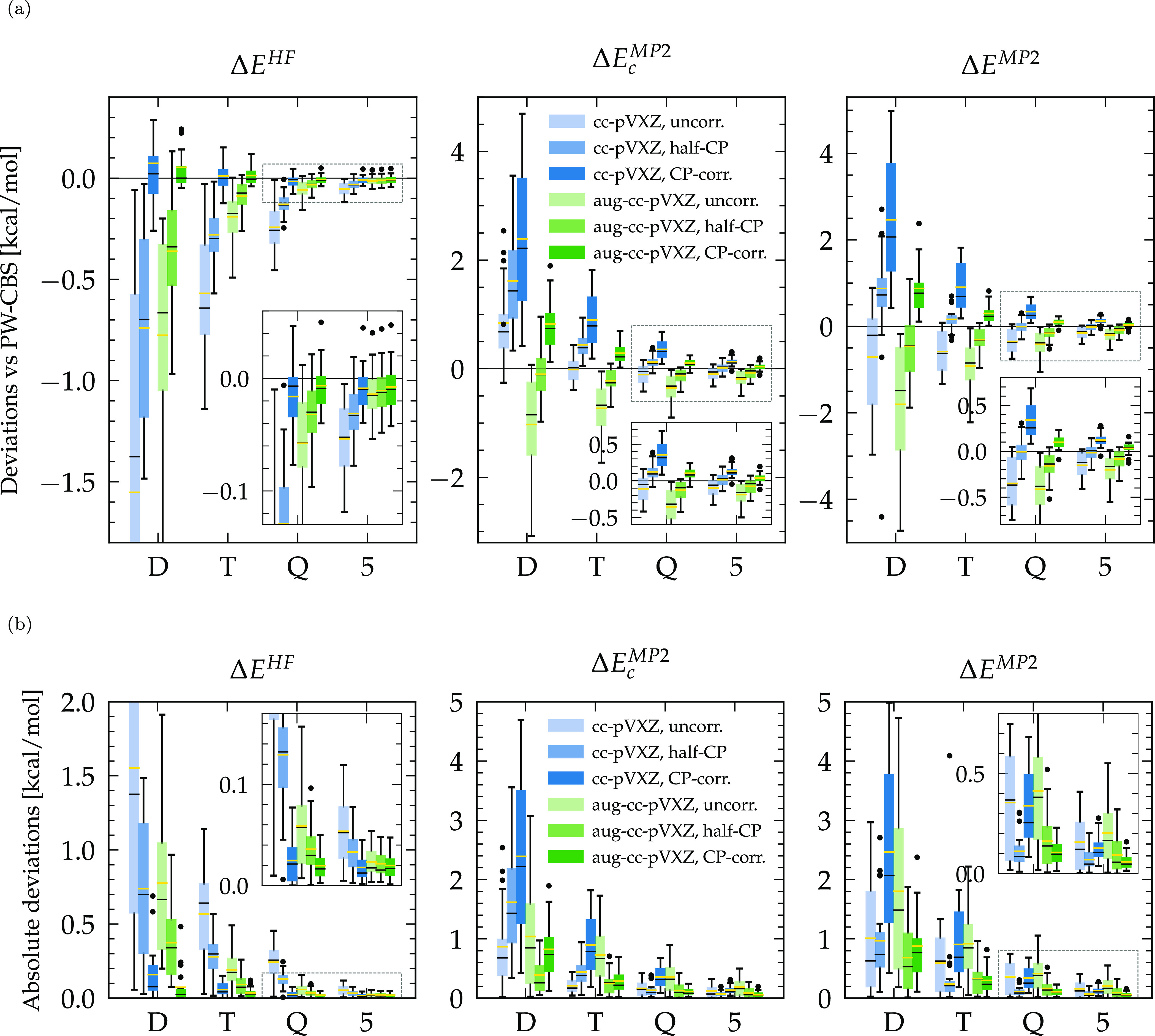
Box plots of the differences Δ*E*_GTO_ – Δ*E*_PW_ between the GTO and PW interaction energies of the S22* test systems. Separate HF and MP2c contributions to the total MP2 energies are given. Signed differences are given in (a) while (b) reports absolute values. Medians are shown as horizontal black lines, and yellow lines stand for the mean signed deviation (MSD) in (a) and the mean absolute error (MAE) in (b), respectively. Dots represent outliers that are located further than 1.5 times the interquartile range from the first and third quartiles (i.e., the limits of the rectangular boxes).

Figure 2 displays the statistics of the differences between the GTO and PW contributions to the MP2 interaction energy. For HF, the CP uncorrected or half-corrected energies converge from below and confirm that the BSSE tends to overbind dimer systems at the HF level. Once the BSSE is removed by the CP correction, HF energies converge faster and from above, which is the expected behavior of a gradual decrease of the (sole) BSIE as the size of the basis set increases.^15^ The augmented basis converges slightly faster than its standard counterpart, certainly due to its larger size and spatial extent for the same cardinal number. When CP-corrected, the HF energies already converged within less than 0.2 kcal/mol for the triple (T) zeta bases. Overall, at each cardinal number, the CP-corrected results obtained with the augmented basis sets provide the best agreement with PWs, as also reported in Table 2. The remarkably small deviations at the HF level between the Q/5 zeta all-electron GTOs and PWs support the fact that the use of pseudopotentials does not cause any spurious differences between the PW (pseudopotential) and GTO (all-electron) results.

**Table 2:** Best Agreement between GTO and PW Interaction Energies of the S22* Test Set at Each Cardinal Number^a^

level	size	set	BSSE corr.	MAE	max dev.
HF	D	non-aug	CP	0.16	0.69
		aug	CP	0.07	0.48
	T	non-aug	CP	0.06	0.15
		aug	CP	0.04	0.12
	Q	non-aug	CP	0.02	–0.08
		aug	CP	0.02	0.05
	5	non-aug	CP	0.02	0.04
		aug	CP	0.02	0.05
MP2	D	non-aug	half-CP	0.97	2.71
		aug	half-CP	0.68	–1.88
	T	non-aug	half-CP	0.24	0.70
		aug	CP	0.30	0.82
	Q	non-aug	half-CP	0.11	0.31
		aug	CP	0.11	0.23
	5	non-aug	half-CP	0.07	–0.20
		aug	CP	0.06	0.16

a Mean absolute errors (MAE) and maximum deviations are in kcal/mol.

The MP2c correlation and, hence, the MP2 energies are more sensitive to the basis set size and slower to converge than HF, with, e.g., MP2 deviations of about 0.7–0.8 kcal/mol for the T zeta bases. This is because the MP2c energy is more prone to the BSIE, which is noticeably exacerbated for the smaller cc-pVDZ and cc-pVTZ bases once CP corrections are applied. Overall, D zeta basis sets do not provide a satisfactory level of convergence, with deviations that might surpass the order of 2 kcal/mol, whatever the augmentation or the BSSE correction. At small D and T cardinalities, the best match with PWs is observed when considering only half of the CP correction with the (aug-)cc-pVDZ or cc-pVTZ bases, respectively (Table 2), which confirms that the accuracy at such levels is mainly due to a fortuitous cancellation of the BSSE, which lowers the energies, and the BSIE, which increases them. Although not formally recommended, this can be exploited for a crude first estimate in the case of a limited computational budget, as provided, for example, by the cc-pVTZ/half-CP combination (with a potential ∼1 kcal/mol error). For the same reason, the match between nonaugmented Q/5 bases and PWs is better with only a half-CP correction. For the augmented bases, however, the same conclusions as for HF hold: the MP2c and MP2 energies are generally too low without a full BSSE correction and approach the CBS PW values from above when corrected. Thanks to this, the overall best agreement between GTOs and PWs is measured on the aug-cc-pV5Z basis with CP correction, which shows a MAE of only 0.06 kcal/mol.

As a result, the gradual convergence of GTOs toward PW values validates both the MP2 implementation in CPMD and the previously proposed extrapolation procedure to compute PW interaction energies in the CBS limit (Section 4.1). Furthermore, our results highlight the importance of diffuse basis functions needed to incorporate the long-range components of the electron correlation in weakly bound systems,^a^ and in this respect, they provide further confirmation of the use of (aug-)cc-pVXZ bases for converging binding/interaction energies with correlated methods, although they were originally designed for the treatment of anions and electron affinities.^19,20,64^

### 4.3 HF/MP2 Energies in PWs Versus Extrapolated GTO Bases

We are now interested in exploring whether the different extrapolations of Scheme 1 for consecutive *X* = D,T,Q,5 cardinal numbers improve or deteriorate the GTO CBS estimates compared to PW energies. When uncorrected for the BSSE, HF, and MP2 interaction energies, they converge nonsystematically and sometimes nonmonotonically because of the varying balance between BSSE and BSIE (as illustrated in Figure S2 or ref (15)). For this reason, GTO extrapolations performed on relative energies yield results that are very similar to or worse than those for absolute total energies. Thus, in what follows, the energy of each subsystem will be extrapolated individually.

**Figure 3:**
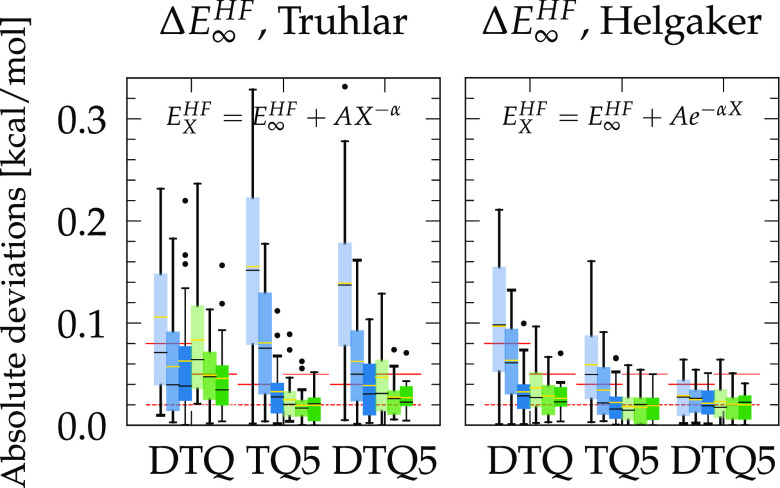
Box plots of the differences |Δ*E*_GTO_^HF^ – Δ*E*_PW_^HF^| between extrapolated GTO and PW HF interaction energies of the S22* test systems. Medians are shown as horizontal black lines, and yellow lines stand for the mean absolute error (MAE). The dashed (solid) red lines correspond to the smallest MAE (maximum deviation) obtained with plain Q and 5 zeta basis sets reported in Table 2. The legend is given in Figure 2. Signed deviations are also provided in Figure S3.

Truhlar (21) and Helgaker (28) treat the HF contribution separately,^b^ and Figure 3 shows how they perform in this regard. Both contain three parameters and require at least three data points for extrapolation. The power expression of Truhlar with D, T, and Q (DTQ) data points generally worsens the deviations from PWs compared with simple Q energies. When including the 5 points (TQ5 and DTQ5), the results are also worse than or comparable to the plain 5 values in terms of MAE and maximum deviation for both nonaugmented and augmented bases. For Helgaker, DTQ points improve the convergence of uncorrected and half-CP Q zeta energies (Figure 2b, Δ*E*^HF^) but slightly deteriorate those that are CP corrected. The TQ5 results are essentially similar to the plain 5 zeta energies, and all extrapolated values are either slightly better or similar when considering all DTQ5 points. Thus, Helgaker (28) applied with the CP correction always gives the best match with PW HF energies, as summarized in Table 3. From this observation, such an exponential expression is the most appropriate for extrapolating HF interaction energies, which are degraded if extrapolated with the scheme of Truhlar. Although its usefulness is rather marginal on relative energies that are essentially converged from the Q zeta level (cf. Table 2), the agreement or small improvement over the nonextrapolated results and the quality of interpolation (Table S3) support the fact that the total (absolute) HF energies can be accurately extrapolated with Helgaker (28).

**Table 3:** Best Agreement between Extrapolated GTO and PW Interaction Energies of the S22* Test Set for Different Fitting Points^a^

points	set	scheme	BSSE	MAE	max dev.	worth?
HF						
DTQ	non-aug	Helgaker	CP	0.03	–0.10	≃
	aug	Helgaker	CP	0.03	–0.07	≃
TQ5	non-aug	Helgaker	CP	0.02	–0.07	≃
	aug	Helgaker	CP	0.02	0.05	≃
DTQ5	non-aug	Helgaker	CP	0.02	–0.05	≃
	aug	Helgaker	CP	0.02	0.04	≃
MP2						
DT	non-aug	Helgaker	half-CP	0.16	–0.38	√
	aug	Helgaker	CP	0.07	0.19	√
TQ	non-aug	Martin4	CP	0.07	0.19	√
	aug	Martin4	CP	0.06	–0.15	√
Q5	non-aug	Martin4	half-CP	0.06	–0.20	≃
	aug	Martin4	CP	0.06	–0.18	≃
DTQ	non-aug	Peterson	CP	0.07	0.19	√
	aug	Helg. (Pet.)	CP	0.05	–0.15	√
TQ5	non-aug	Mart.4 (Pet.)	CP	0.06	–0.16	√
	aug	Mart.4 (Pet./Fel.)	CP	0.06	–0.16	≃
DTQ5	non-aug	Helg. (Pet.)	CP	0.07	0.18	≃
	aug	Helgaker	CP	0.05	–0.16	≃

a Mean absolute errors (MAE) and maximum deviations are in kcal/mol. Worth indicates that the extrapolated energies are statistically closer to the CBS PW values than the corresponding direct results obtained at the highest extrapolation point (cf. Table 2).

With respect to MP2 interaction energies, the results show that a majority of GTO extrapolations generally induce larger deviations from PW values than results directly obtained with the highest *X* point in the fitting sequence. This is particularly the case for CP-corrected energies, for which extrapolations are expected to perform well on the reminiscent BSIE effects, and happens for the expressions of Truhlar (22), Martinα (25), Wilson35 (26), Wilson45 (27), Varandas34 (30), Varandas3-fit (31), and Var.34-fit (32). These schemes can therefore be invalidated outright to accurately estimate GTO energies in the CBS limit and are left in the Supporting Information for the interested reader’s discretion (Figures S5–S7).

**Figure 4:**
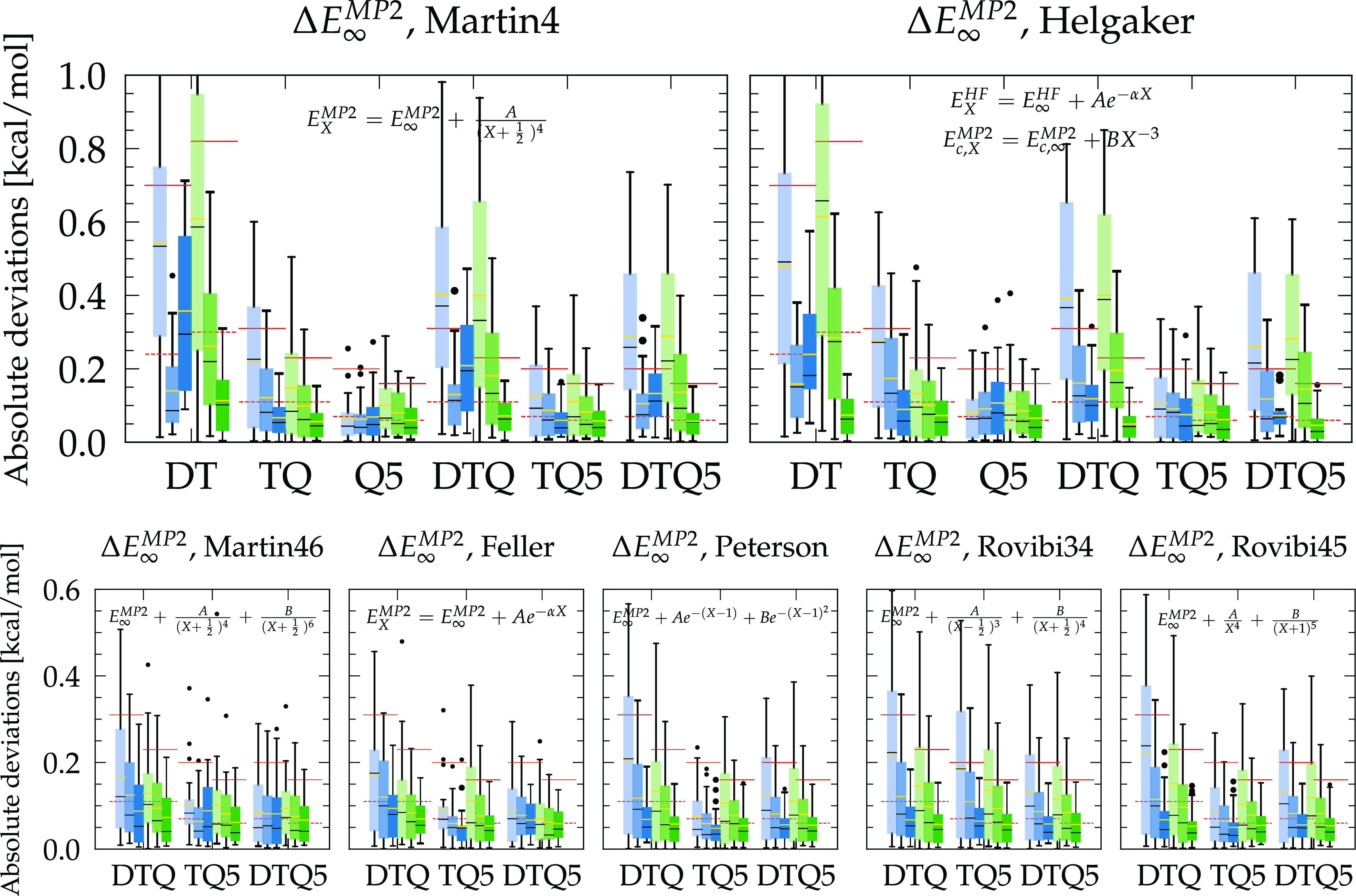
Box plots of the differences |Δ*E*_GTO_^MP2^ – Δ*E*_PW_^MP2^| between extrapolated GTO and PW MP2 interaction energies of the S22* test systems. Medians are shown as horizontal black lines, and yellow lines stand for the mean absolute error (MAE). The dashed (solid) red lines correspond to the smallest MAE (maximum deviation) obtained with plain T, Q, or 5 zeta basis sets, respectively, as reported in Table 2. The legend is given in Figure 2. Signed deviations are also provided in Figure S4.

The remaining extrapolations are plotted in Figure 4. Martin4 (23) and Helgaker (29) have the advantage of extrapolating GTO energies from two points only, although for the latter, the exponential form of the HF energy requires three parameters, but HF calculations are orders of magnitude cheaper and converge faster than MP2 (as seen previously). Based on DT points, Helgaker provides a better agreement with PWs in terms of MAE and maximum deviation, especially for the aug-cc-pVTZ/CP combination with a MAE (max dev.) of 0.3 (0.8) kcal/mol (Table 2), which reduces to 0.07 (0.19) kcal/mol when extrapolating with DT data points (Table 3). If the available points are TQ instead, the energies obtained by Martin4 are globally closer to the PW values than those of Helgaker, which is also the case for Q5 points, although in these cases, no extrapolation outperforms the aug-cc-pV5Z/CP calculations and their MAE (max dev.) of 0.06 (0.16) kcal/mol (Table 2).

If DTQ points are calculated, Helgaker performs better than Martin4 for both augmented and nonaugmented bases coupled with the CP correction and provides energies that are more convergent than if kept at the Q level only. However, when resorting to nonaugmented basis sets with CP correction, the three-parameter schemes Martin46 (24), Feller (19), and Peterson (20) provide even smaller deviations than Helgaker, with Peterson surpassing the others. Because Martin4 and Helgaker are based on leading orders at large *X* (cf. Section 2.3.1), those are likely to deteriorate when considering the smallest cc-pVDZ basis set in the extrapolation sequence.^26^ Indeed, Martin4 and Helgaker in general produce slightly lower energies with respect to the PW references, but these deviate more widely from above when *D* points are considered (Figure S4). Thus, the fact that Peterson is the most appropriate for the nonaugmented basis with DTQ points appears quite coincidental and may also result from the lack of diffuse functions that causes additional BSIE, not related to the description of the Coulomb cusp but rather due to long-range effects which cannot be fully corrected for by GTO extrapolations.^15^

Finally, extrapolating from the TQ5 points with Martin4 and Peterson gives similar smallest deviations when the energies are CP-corrected, and the same applies to Helgaker and Peterson when using all DTQ5 points, but Helgaker performs somewhat better for the augmented sets. Note that the extrapolations that include the (aug-)cc-pV5Z data point do not further improve the interaction energies (Table 3), with an average MAE (max dev) against PWs of 0.06 (0.17) kcal/mol that is comparable to the 0.07 (0.18) kcal/mol for the nonextrapolated results (Table 2). This remaining difference is discussed in Section 4.5.

To summarize, our results demonstrate that the extrapolated GTO energies are always closer to PW reference values when the CP correction is used to tackle the BSSE with sets that are augmented by diffuse functions (Table 3). For nonaugmented bases, the interplay between the BSIE and some residual BSSE can make the Helgaker and Martin4 two-point extrapolations perform fortuitously better in conjunction with the half-CP correction. The empirical Peterson scheme surprisingly provides interaction energies that are very close to the PW results, comparable to Helgaker or Martin4, but the latter two appear to be more robust candidates because of their theoretical foundations and the fact that they depend on only two parameters. To confirm this, the same analysis has been carried out with the omission of four outlier systems (the two uracil, benzene–water, and T-shaped indole benzene dimers for which the aug-cc-pV5Z/CP interaction energies are already lower than the CBS PW values). For this smaller test set, Feller occasionally beats Peterson, but Helgaker and Martin4 perform consistently better too. Therefore, if the extrapolation sequence includes the D zeta level, then it is suggested to use the Helgaker (28)(29) scheme on aug-cc-pVXZ CP-corrected energies in order to obtain the most accurate estimates in the CBS limit. If not, then the Martin4 (23) expression is recommended.

### 4.4 Other GTO Extrapolations

Motivated by the best agreements found so far, as well as the general expression of eq 36, we have tested all possible extrapolations in the form of
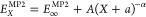
44
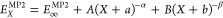
45with α, β = 3, 4, 5, 6 and  to investigate whether different schemes could universally improve the remaining deviations reported in Table 3. For the first eq 44, no combination gives overall better results, whether for the augmented or nonaugmented bases, reinforcing the recommendation of Helgaker (29) for DT points only and Martin4 (23) for TQ or Q5 pairs. If three points are available, however, two new expressions stand out as providing very similar or lower deviations than those of Helgaker and Martin4. We call them, from now on, Rovibi34 and Rovibi45 defined by
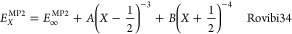
46
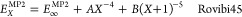
47whose results are also reported in Figure 4 and Table 4 for comparison.

**Table 4:** Best Agreement between Rovibi Extrapolations and PW Interaction Energies for the S22* Test Set^a^

points	set	scheme	BSSE corr.	MAE	max dev.	worth?
MP2						
DTQ	non-aug	Rovibi34	CP	0.07	0.18	√
	aug	Rovibi34	CP	0.06	–0.15	√
TQ5	non-aug	Rovibi34	CP	0.06	0.16	√
	aug	Rovibi34	CP	0.06	–0.15	≃
DTQ5	non-aug	Rovibi34	CP	0.06	–0.15	√
	aug	Rovibi34	CP	0.06	–0.15	≃
DTQ	non-aug	Rovibi45	CP	0.07	0.22	√
	aug	Rovibi45	CP	0.05	–0.15	√
TQ5	non-aug	Rovibi45	CP	0.05	–0.16	√
	aug	Rovibi45	CP	0.05	–0.15	≃
DTQ5	non-aug	Rovibi45	CP	0.06	0.15	√
	aug	Rovibi45	CP	0.05	–0.15	≃

a Mean absolute errors (MAE) and maximum deviations are in kcal/mol. Worth indicates that the extrapolated energies are statistically closer to the CBS PW values than the corresponding direct results obtained at the highest extrapolation point (cf. Table 2).

Such laws indicate that the −3 and −4 orders are indeed good leading candidates and that terms of higher orders may also be significant. If some rational explanation were to be found, Rovibi34 (46) is compatible with contributions resulting from the principal expansion proposed by Helgaker^1,22^ (power −3) and those of the highest angular momentum *L* present in the basis set from the partial wave expansions put forward by Carroll,^80^ Hill,^81^ and Kutzelnigg.^79^ On the other hand, Rovibi45 (47) suggests that an additional order to Martin4 improves the extrapolation and that the (minus) third order does not dominate. For both, the X-shifts reflect not only the balance between the orders but also between the basis functions that have *L* = *X* – 1 for H and *L* = *X* for C, N, and O atoms in the systems studied here. Based on these considerations and because the leading order of Martin4 (−4) was motivated empirically by comparison with experimental atomization energies of small molecules,^25,82,83^ the Rovibi34 scheme seems more formally justified.

Up to this point, only the relative energies extrapolated to the CBS limit have been compared to PW results, but the quality of the GTO extrapolations can also be assessed by how faithfully they reproduce the single data points. Averaged on all systems, the fitting curves of Rovibi34 and Rovibi45 show a MAE relative to the data points of no more than 0.5 kcal/mol (Table S3), while the latter lies between 3.8 and 7.6 kcal/mol for Helgaker and Martin4. As a reminder, these errors refer to the total (absolute) energies of the dimers and monomers that were extrapolated individually. Hence, Rovibi34 and Rovibi45 not only provide interaction energies close to the PWs in the CBS limit but are also capable of interpolating total energies well. In this respect, Rovibi34 performs best with a MAE of 0.15–0.3 kcal/mol, compared to 0.3–0.5 kcal/mol for Rovibi45. We also stress that the double exponential scheme of Peterson, although purely empirical, shows even smaller fitting MAEs of ∼0.1 kcal/mol (Table S3) and provides deviations against PWs that are similar to those of Rovibi34 (Figure 4). Consequently, our results do not disprove it as a good extrapolation law. However, based not only on agreement with PWs and the ability to interpolate GTO energies but also on theoretical indications, it appears that Rovibi34 (46) is likely the best global choice when resorting to three-/four-point extrapolations (DTQ, TQ5, and DTQ5) to the CBS limit.

### 4.5 System Size Dependency

**Figure 5:**
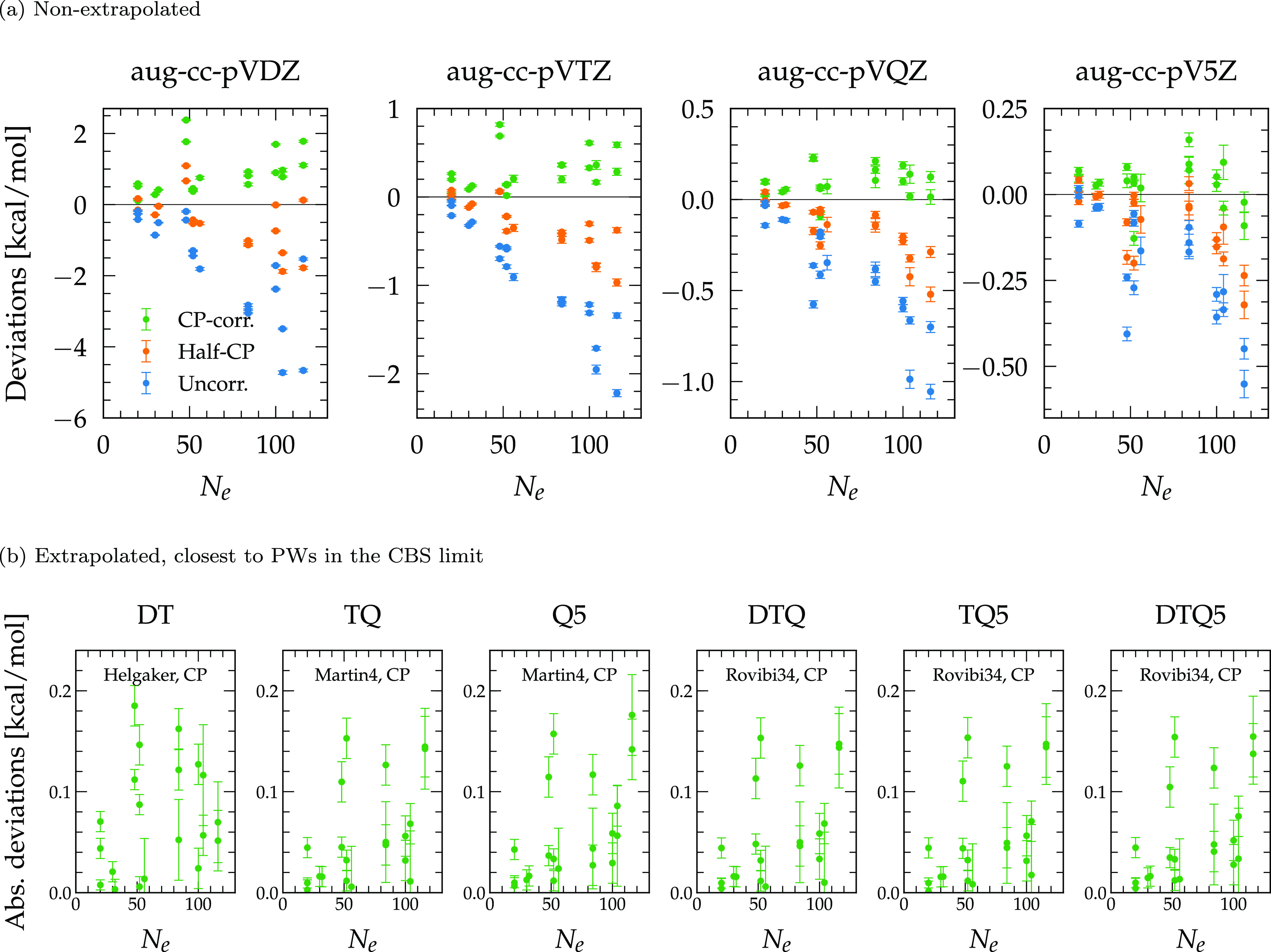
Deviations between the aug-cc-pVXZ and PW MP2 interaction energies as a function of the number of electrons *N*_e_ in the dimer system. (a) For plain basis set results and (b) for energies extrapolated to the CBS limit with the best schemes of Tables 3 and 4.

Finally, whatever the effort in order to match GTOs with PW results, via CP correction, basis set augmentation, or extrapolation, one notices that the best MP2 errors do not reach MAEs lower than 0.05 kcal/mol with maximum differences of at least 0.15 kcal/mol (Tables 2–4). While this might at first be taken as an indication that interaction energies are essentially converged at the aug-cc-pV5Z/CP level within an 0.05 kcal/mol numerical accuracy, further analysis instead reveals that residual discrepancies occur because GTO results tend to further deviate from PWs as the system size increases. Figure 5a shows indeed that GTO interaction energies that are not corrected for the BSSE are lower than those from PWs and that the BSSE tends to become larger with an increasing number of electrons in the system. Interestingly, assuming that the CP correction removes the majority of the BSSE, only the BSIE remains and, in turn, increases with the system size. Hence, although the size of a GTO basis grows with the number of atoms, the incomplete coverage (lack of completeness) of this expansion leads to a smaller and smaller correction of the BSIE with an increasing system size. In other words, for a given GTO basis set, its capacity to capture the (correlation) energy decreases as the size of the system increases. Such a size inconsistency is even more pronounced for the smaller cc-pVXZ bases (Figure S9a). In the limits of large bases and systems, however, the occurrence of linear dependencies can further interfere with this behavior.

Once extrapolated, the GTO CBS estimates follow a similar trend with larger (absolute) differences attributed to larger numbers of electrons *N*_e_ (Figures 5b and S9b), thus questioning the agreement between GTOs and PWs in the limit of (very) large systems. Note that such a difference applies to all promising extrapolations found in this work (Figures S10 and S11). The reasons can be multiple and arise from a combination of the BSSE, the accuracy of the extrapolation scheme, and the intrinsic nature of the basis functions. Nevertheless, as observed earlier (Section 4.2), the CP correction seems adequate to eliminate the BSSE so that the last two factors will dominate, which are directly linked to the BSIE. Let us recall that the initial motivation behind the extrapolation schemes is to cope with the electron–electron cusp that hampers the basis set convergence.^1,15^ Therefore, although not firmly established by our results, the fact that extrapolated energies deviate from PWs could indicate a residual completeness mismatch between GTOs and PWs in the limit of large systems, originating rather from an incomplete or linearly dependent (overcomplete) GTO description of the wave function to capture the long-range (polarization and dispersion) contributions to the correlation energy. This encourages further comparison of atom-centered bases against other basis sets, either explicitly correlated, PWs, or purely numerical, in the calculation of the correlation energies of large systems.

## 5 Conclusions and Outlook

The main motivation for this work was to analyze the effects of the basis sets on correlated energies. To this end, the MP2 interaction energies of 20 complexes belonging to the S22 test set were computed in the most common Gaussian-type correlation-consistent bases as well as in PWs, for which we implemented the MP2 method in the CPMD plane-wave pseudopotential package. Although more computationally demanding at such system scales, PW calculations have been made accessible on conventional computing architectures through an extrapolation protocol involving both the virtual space orbitals and the supercell volume, ultimately requiring no more than a few days per molecule on several high-memory compute nodes.

By comparing atom-centered interaction energies with PW results, we established that both basis set types provide consistent values, especially when the CP correction eliminates the BSSE from the former. Indeed, (aug-)cc-pVXZ relative energies generally converge toward PW values, free of BSSE, for progressive enlargements of *X* = D,T,Q,5, and differ by less than 1 kcal/mol for *X* ≥ T. However, the slower convergence of the cc-pVXZ bases makes their agreement with PWs occasionally better, with only half of the CP correction due to a fortuitous error cancellation between the BSSE and their BSIE. Overall, the aug-cc-pV5Z basis set with the CP correction provides the closest interaction energies within 0.16 kcal/mol to the fully converged PW results. This demonstrates the benefits of diffuse functions in the description of long-range interactions as occurring in weakly bound systems, although their faster energy convergence may slow down for stronger (covalent) interactions.^71^

Hence, based on the agreement with PW results at the CBS limit, theoretical foundations, and interpolation capabilities, we can confidently make the following recommendations for the extrapolation of (aug-)cc-pV[D,T,Q,5]Z correlated energy sequences to the CBS limit:

- Use the CP correction for interaction/binding energies.
- Resort to the aug-cc-pVXZ bases if long-range effects are sizable.
- Extrapolate total energies separately according to
  - (D)TQ5 points and
  - DTQ points and
  - Q5 points and
  - TQ points and
  - DT points and *A X*^–3^

where the choice of the extrapolation points (and basis augmentation) is left to the practitioner since these will depend on the computational budget and the problem/software at hand. However, note that in principle, the higher the point in the sequence, the more accurate the extrapolated value is, and the DT scheme should rather be taken as a first rough estimate. In practice, the universal application of such a procedure across correlated wave function methods (MP2, CCSD, CCSD(T), ...) has been widely accepted.^15,22,23,25,26,29,70,76,83^

Finally, getting the electron correlation in the CBS limit relies on the saturation of the one-electron basis, whose basis functions should adequately span short distances and be flexible enough to incorporate long-range components of the wave function. The latter are directly linked to the delocalized nature of the virtual states that contribute to the correlation energy and therefore necessitate balanced and complete space coverage. In that sense, PWs are capable of capturing high-lying (continuum-like) states as well as localized occupied states, at the cost of a sufficiently large cutoff energy.^47^ When compared to PWs, we noticed that the ability of the correlation-consistent bases to cope with the BSIE decreases as the number of electrons increases, therefore questioning the capability of localized basis sets to recover most of the correlation energy as the system size increases. PW, explicitly correlated, or numerical basis calculations on larger systems would confirm (or refute) this statement, but their computational overhead seems to compromise their application for the time being. It is therefore not excluded that, with the improvement of wave function-based methods, the precision of correlated energies becomes comparable to the basis set errors, such that the nature of the basis or its extrapolation to the CBS limit ultimately becomes dominant and leads to noticeable deviations that exceed chemical accuracy (1 kcal/mol) for larger molecules.^93^

## Supporting Information Available

Implementation details of the PW MP2 correlation energy in CPMD; energy convergence against the PW wave function energy cutoff; energy convergence versus the box volume for different treatments of the electrostatic couplings in the PW periodic setup; PW and (aug-)cc-pV5Z S22* interaction energies; additional deviations between GTO and PW interaction energies against cardinal numbers and the number of electrons in the complexes; and figures of merit for the interpolation quality of various GTO extrapolations (PDF)

## Notes

The development of this work is intended to be available in a future release of CPMD.^35^ Data, extrapolation, and analysis scripts are provided on Zenodo at https://doi.org/10.5281/zenodo.7838778.

## Footnotes

a For stronger (e.g., covalent) interactions, however, diffuse augmentation was sometimes found to hamper the basis-set convergence of correlation energies.^71^

b Note that Martinα (25) was also proposed for HF energies and yields identical conclusions to Truhlar (21) (not reported).
