# Supplementary material for: Plane Waves Versus Correlation-Consistent Basis Sets: A Comparison of MP2 Non-Covalent Interaction Energies in the Complete Basis Set Limit
Source: J Chem Theory Comput. 2023 Dec 4;19(24):9211–27. doi: 10.1021/acs.jctc.3c00952 (PMC10753812; doi:10.1021/acs.jctc.3c00952)
Supplement: Supplementary file 1 — ct3c00952_si_001.pdf [file ct3c00952_si_001.pdf]

Plane Waves versus Correlation-Consistent Basis Sets: A Comparison of MP2 Non-Covalent Interaction Energies in the Complete Basis Set Limit

Justin Villard[1], Martin P. Bircher[2], and Ursula Rothlisberger[1]*

[1]*Laboratory of Computational Chemistry and Biochemistry, Institute of Chemical Sciences and Engineering, École Polytechnique Fédérale de Lausanne (EPFL), CH-1015 Lausanne, Switzerland*
[2]*Computational and Soft Matter Physics, Universität Wien, A-1090 Wien, Austria*

E-mail: ursula.roethlisberger@epfl.ch

Supporting Information

MP2 implementation in CPMD

Algorithm 1 presents the details of the calculation of $E_{c,n}^{\mathrm{MP2}}$ (eq 9 of the main text) that uses the existing mixed distributed/shared (MPI/OpenMP) parallelization strategy[31,32] of CPMD (CP_GROUPS are currently not supported). The work load is divided into blocks. Within a block, a list of summand indices is created. Then, the partial two-electron integrals are saved in an array, and summed across tasks only once the loop has been completed. This allows for the OpenMP parallelization of the outermost loop, leading to a much smaller shared-memory overhead from thread creation. Additionally, inter- and intra-task-communication becomes much cheaper, as one big array is distributed once per block, instead of a single number being distributed for every $ijab$ tuple. This saves a lot of overhead. Then, the summed partial integrals are used to calculate the final MP2c energy.

Note that at the Γ-point, the orbital coefficients can be chosen to be real, introducing the symmetry $\tilde{\phi}_{i,a}(\mathbf{G}) = \tilde{\phi}_{i,a}^{*}(-\mathbf{G})$ which can be exploited to speed-up the code.

In order to extrapolate the MP2 correlation energy at the basis set limit (eq 11), $E_{c,n}^{\mathrm{MP2}}$ values are printed out whenever n is incremented by b_{incr} virtual orbitals until $n_{\max}$. This increment is user-defined; the larger the value, the more efficient the calculation, which however becomes more memory intensive and provides less extrapolation points. In this work, $b_{incr} = 100$ was found as a good compromise. In addition, $n_{\max}$ is defined sufficiently large as to reach a reliable extrapolation regime according to eq 11 that is valid at high virtual index. Values between $n_{\max} = 10000$-20000 were used herein for relative energies. A RESTART mechanism is available in order to diagonalize supplementary virtual orbitals and continue the computation of $E_{c,n}^{\mathrm{MP2}}$ for larger $n_{\max}$.

This implementation corresponds to the one used in our recent study on the acceleration of the MP2c energy calculation by stochastic sampling of the virtual space integrands based on Monte Carlo summation.[47] In that case, the stochastic sampling is carried out by selecting which $ijab$ tuples will be effectively included in the *list* for later evaluation and proper renormalization.

```
Input  : $n_{\max}$, $E_{cut}^{\rho_{ia}}$, virtual block increment $b_{incr}$
Output: $E_{c,n}^{\mathrm{MP2}}$ for $n \bmod b_{incr} = 0$
Wavefunction optimization with HF $\rightarrow E^{\mathrm{HF}}$, $\tilde{\phi}_i(\mathbf{G})$, $\varepsilon_i$;
Diagonalization of the $n_{\max}$ lowest virtuals $\rightarrow \tilde{\phi}_a(\mathbf{G})$, $\varepsilon_a$;
/* The G vectors are distributed among the MPI tasks for all $i, a$ orbitals */
$\phi_{i,a}(\mathbf{r}) \leftarrow \mathrm{FFT}^{-1}_{E^{\phi}_{cut}} [\tilde{\phi}_{i,a}(\mathbf{G})]$;
for $i \leftarrow 1$ to $N_{occ}$ do
    for $a \leftarrow 1$ to $n_{max}$ do
        $\rho_{ia}(\mathbf{r}) \leftarrow \phi_i^*(\mathbf{r})\phi_{a+N_{\mathrm{occ}}}(\mathbf{r})$;
        $\rho_{ia}(\mathbf{G}) \leftarrow \mathrm{FFT}_{E_{cut}^{\rho_{ia}}}[\rho_{ia}(\mathbf{r})]$;
    end
end
$E_{c,n}^{\mathrm{MP2}} \leftarrow 0$, $n_{blocks} \leftarrow \mathrm{ceil}(n_{\max}/b_{incr})$;
for $n_{low} \leftarrow 1$ to $n_{blocks}$ by $b_{incr}$ do
    /* Block contribution from adding $b_{incr}$ new virtuals, create list of contributing ijab tuples and make use of
       the symmetries of the integrals and MP2c summands */
    $n_{high} \leftarrow \min(n_{low} + b_{incr} - 1, n_{\max})$;
    for $i \leftarrow 1$ to $N_{occ}$ do
        for $j \leftarrow i$ to $N_{occ}$ do
            for $a \leftarrow n_{low}$ to $n_{high}$ do
                for $b \leftarrow a$ to $n_{high}$ do
                    $ijab \leftarrow (i, j, a, b)$;
                    $list$.append($ijab$);
                end
            end
            for $a \leftarrow 1$ to $n_{low}$ do
                for $b \leftarrow n_{low}$ to $n_{high}$ do
                    $ijab \leftarrow (i, j, a, b)$;
                    $list$.append($ijab$);
                end
            end
        end
    end
    $\langle ij|ab \rangle \leftarrow$ Array(size: $list$.size, elements: 0);
    $\langle ij|ba \rangle \leftarrow$ Array(size: $list$.size, elements: 0);
    /OMP parallelized loop/;
    forall $ijab \in list$ do
        forall $\mathbf{G}$ defined by $E_{cut}^{\rho_{ia}}$ do
            /* Within a MPI task */
            $\langle ij|ab \rangle [ijab] \leftarrow \langle ij|ab \rangle [ijab] + \Phi(\mathbf{G})\rho_{ia}(\mathbf{G})\rho_{jb}(\mathbf{G})$;
            $\langle ij|ba \rangle [ijab] \leftarrow \langle ij|ba \rangle [ijab] + \Phi(\mathbf{G})\rho_{ib}(\mathbf{G})\rho_{ja}(\mathbf{G})$;
        end
    end
    MPI_SUM $\langle ij|ab \rangle [1, \ldots, list.\mathrm{size}]$ across all MPI tasks;
    MPI_SUM $\langle ij|ba \rangle [1, \ldots, list.\mathrm{size}]$ across all MPI tasks;
    /OMP parallelized loop, + reduction/;
    forall $ijab \in list$ do
        /* Factors for symmetries */
        if $i = j$ and $a = b$ then
            $f \leftarrow 1$ ;
        else
            if $i = j$ or $a = b$ then
                $f \leftarrow 2$ ;
            else
                $f \leftarrow 4$ ;
            end
        end
        $E_{c,n}^{\mathrm{MP2}} \leftarrow E_{c,n}^{\mathrm{MP2}} + \frac{f}{\Omega^2} \frac{(\langle ij|ab \rangle [ijab])^2 - \langle ij|ab \rangle [ijab] \langle ij|ba \rangle [ijab] + (\langle ij|ba \rangle [ijab])^2}{\varepsilon_i + \varepsilon_j - \varepsilon_a - \varepsilon_b}$
    end
    print $E_{c,n}^{\mathrm{MP2}}$ $(n = n_{high})$ for extrapolation at consecutive $b_{incr}$
end
```

Algorithm 1: Pseudocode for the calculation of the MP2c energy in CPMD.[35]

Table S1: HF and MP2c contributions to the MP2 interaction energy for some S22 systems and wavefunction cutoff energy E^{ϕ}_{cut}. Energies are in [kcal/mol]. $r_{x,y,z}$ are the respective x, y, z ratios of the orthorhombic supercell dimensions with respect to the HF electron density measured at an isosurface of 0.002 a.u., while Ω is the volume of the supercell. σ_c^{MP2} corresponds to the standard deviation of $\Delta E_c^{\mathrm{MP2}}$ values extrapolated on different fitting ranges according to eq 38. The density cutoff energy is $E^{\rho}_{cut} = 4E^{\phi}_{cut}$ and its analogue for the MP2c pair densities is $E^{\rho_{ia}}_{cut} = E^{\phi}_{cut}$.

S22 system	r_x r_y r_z	Ω [Å^3]	E^{ϕ}_{cut} [Ry]	ΔE^{HF}	$\Delta E_c^{\mathrm{MP2}}$	ΔE^{MP2}	σ_c^{MP2}
$(NH_3)_2$	2.0 2.0 2.0	987.84	150	-1.428	-1.763	-3.191	0.004
			180	-1.429	-1.762	-3.191	0.002
	2.0 2.9 2.9	1822.02	150	-1.430	-1.752	-3.182	0.006
			180	-1.430	-1.751	-3.181	0.006
	2.0 3.6 3.5	2744.00	150	-1.430	-1.758	-3.188	0.011
	(*cubic*)		180	-1.431	-1.751	-3.182	0.011
$(H_2O)_2$	1.7 1.9 2.2	648.86	150	-3.618	-1.354	-4.972	0.004
			160	-3.622	-1.347	-4.969	0.004
			180	-3.638	-1.345	-4.983	0.004
	2.0 2.0 2.0	686.16	150	-3.599	-1.358	-4.957	0.003
			180	-3.618	-1.344	-4.962	0.001
Formamide	1.4 1.4 1.4	539.82	150	-11.768	-3.658	-15.426	0.004
			180	-11.821	-3.626	-15.447	0.006
	2.0 2.0 2.0	1573.83	150	-12.166	-3.549	-15.715	0.008
			180	-12.221	-3.499	-15.720	0.005
PD benzene	1.6 1.8 1.7	1550.20	150	6.207	-10.751	-4.543	0.012
			180	6.206	-10.750	-4.544	0.012
Benzene · H_2O	2.0 2.0 2.0	2954.17	150	-0.924	-2.444	-3.367	0.010
			180	-0.929	-2.428	-3.357	0.013

(a) Monomer (b) Dimer

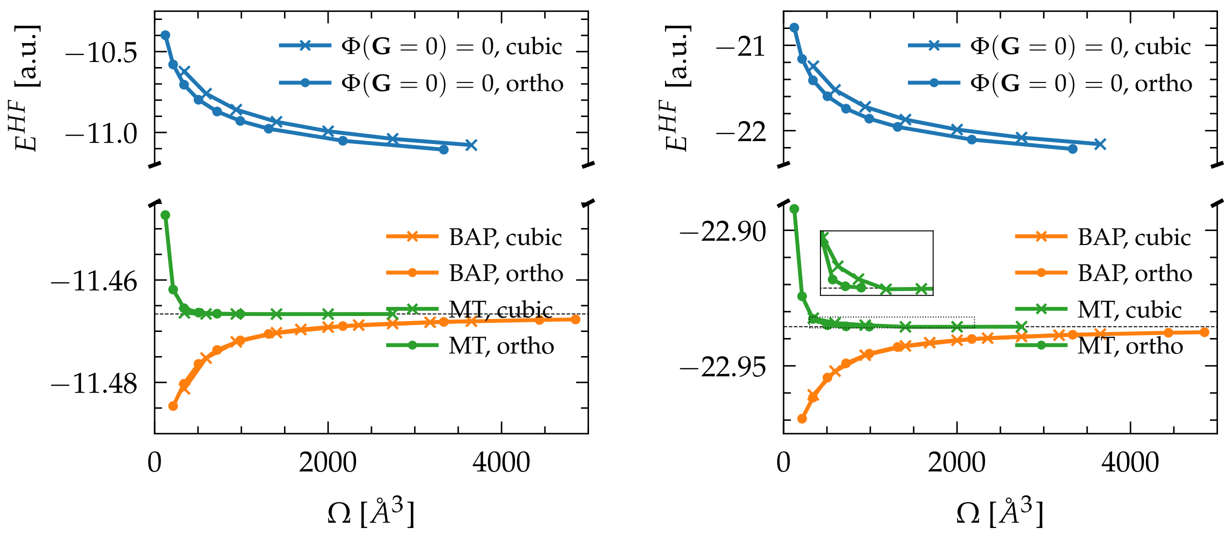

Figure S1: HF energy of the (a) NH_3 monomer and (b) $(NH_3)_2$ dimer for different exchange (Coulomb) potentials. Ω is the volume of expanding cubic or orthorombic supercells around the dimer electron density.

Table S2: MP2 interaction energies in [kcal/mol] of the S22* test systems, uncorrected and with CP correction for the 5 zeta GTO basis sets. The PW values are given with the standard deviation σ resulting from the two consecutive extrapolations: with respect to the virtual orbitals (eq 38) and the supercell volume (eq 43). Mean signed deviations (MSD) and mean absolute errors (MAE) against PWs are indicated, respectively for each dominant interaction type and over all systems.

S22* test set		cc-pV5Z		aug-cc-pV5Z		PWs
No.	complex	uncorr.	CP-corr.	uncorr.	CP-corr.	CBS $\pm 1\sigma$
Hydrogen-bonded						
1	$(NH_3)_2$	-3.21	-3.08	-3.17	-3.12	-3.19 $\pm$ 0.01
2	$(H_2O)_2$	-5.14	-4.85	-5.04	-4.90	-4.95 $\pm$ 0.01
3	Formic acid dimer	-18.74	-18.22	-18.78	-18.33	-18.37 $\pm$ 0.02
4	Formamide dimer	-15.94	-15.51	-15.96	-15.64	-15.72 $\pm$ 0.01
5	Uracil dimer	-20.57	-20.10	-20.64	-20.21	-20.19 $\pm$ 0.03
6	2-pyridoxine · 2-aminopyridine	-17.54	-17.08	-17.61	-17.20	-17.25 $\pm$ 0.02
		-0.24	*0.14*	*-0.25*	*0.04*	*MSD*
		0.24	*0.14*	*0.26*	*0.05*	*MAE*
Predominant dispersion						
8	$(CH_4)_2$	-0.48	-0.46	-0.51	-0.49	-0.50 $\pm$ 0.01
9	$(C_2H_4)_2$	-1.58	-1.50	-1.63	-1.56	-1.59 $\pm$ 0.01
10	Benzene · CH_4	-1.84	-1.75	-1.90	-1.79	-1.84 $\pm$ 0.01
11	Parallel-displaced benzene dimer	-5.06	-4.78	-5.16	-4.90	-5.06 $\pm$ 0.02
12	Pyrazine dimer	-6.96	-6.67	-7.09	-6.83	-6.92 $\pm$ 0.02
13	Uracil dimer	-11.32	-10.78	-11.46	-11.00	-10.91 $\pm$ 0.04
14	Stacked indole · benzene	-8.27	-7.85	-8.38	-8.01	-8.10 $\pm$ 0.05
		-0.08	*0.16*	*-0.17*	*0.05*	*MSD*
		0.09	*0.16*	*0.17*	*0.08*	*MAE*
Mixed complexes						
16	Ethene · ethine	-1.66	-1.61	-1.71	-1.64	-1.67 $\pm$ 0.01
17	Benzene · H_2O	-3.72	-3.42	-3.64	-3.50	-3.37 $\pm$ 0.02
18	Benzene · NH_3	-2.73	-2.57	-2.74	-2.63	-2.66 $\pm$ 0.01
19	Benzene · HCN	-5.19	-5.05	-5.29	-5.11	-5.13 $\pm$ 0.04
20	T-shaped benzene dimer	-3.71	-3.53	-3.80	-3.59	-3.66 $\pm$ 0.04
21	T-shaped indole · benzene	-7.13	-6.83	-7.22	-6.92	-6.88 $\pm$ 0.02
22	Phenol dimer	-7.91	-7.56	-7.98	-7.66	-7.69 $\pm$ 0.02
		-0.14	*0.07*	*-0.19*	*0.00*	*MSD*
		0.15	*0.09*	*0.19*	*0.05*	*MAE*
		-0.15	0.12	-0.20	0.03	MSD
		0.16	0.13	0.20	0.06	MAE

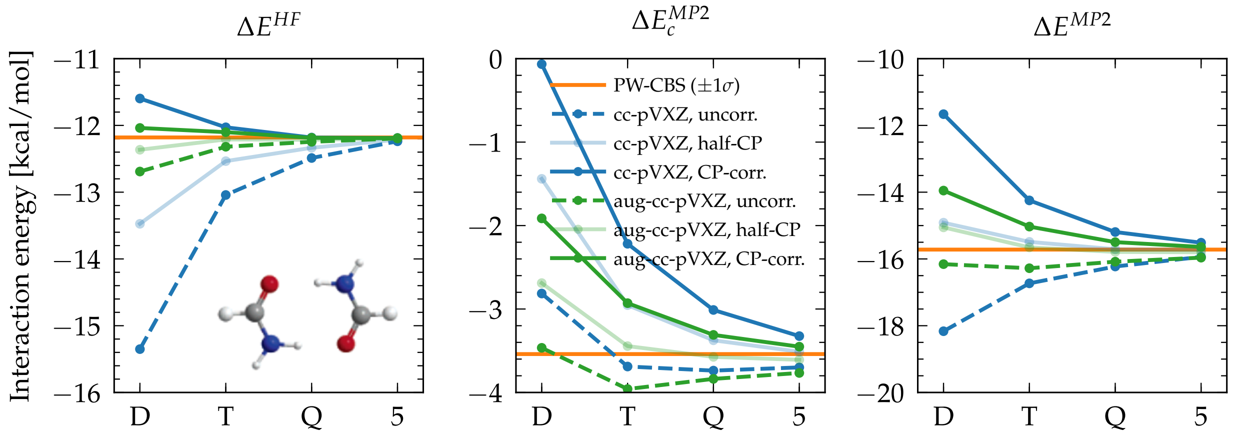

Figure S2: Formamide dimer - Convergence of the HF and MP2c energy contributions to the total MP2 interaction energy for the (aug-)cc-pVXZ basis sets and different treatments of the BSSE. The CBS PW value is also indicated.

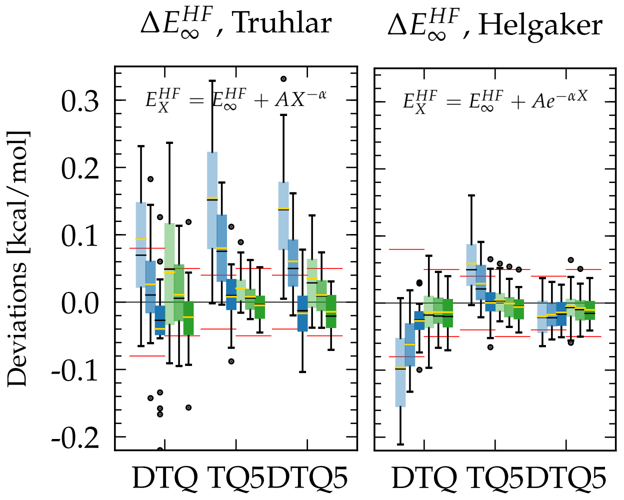

Figure S3: Box plots of the differences $\Delta E^{\mathrm{HF}}_{\mathrm{GTO}} - \Delta E^{\mathrm{HF}}_{\mathrm{PW}}$ between extrapolated GTO and PW HF interaction energies of the S22* test systems. Medians are shown as horizontal black lines and yellow lines stand for the mean signed deviation (MSD). The solid red lines correspond to the smallest maximum deviation obtained with plain Q and 5 zeta basis sets reported in Table 2. The legend is given in Figure 2.

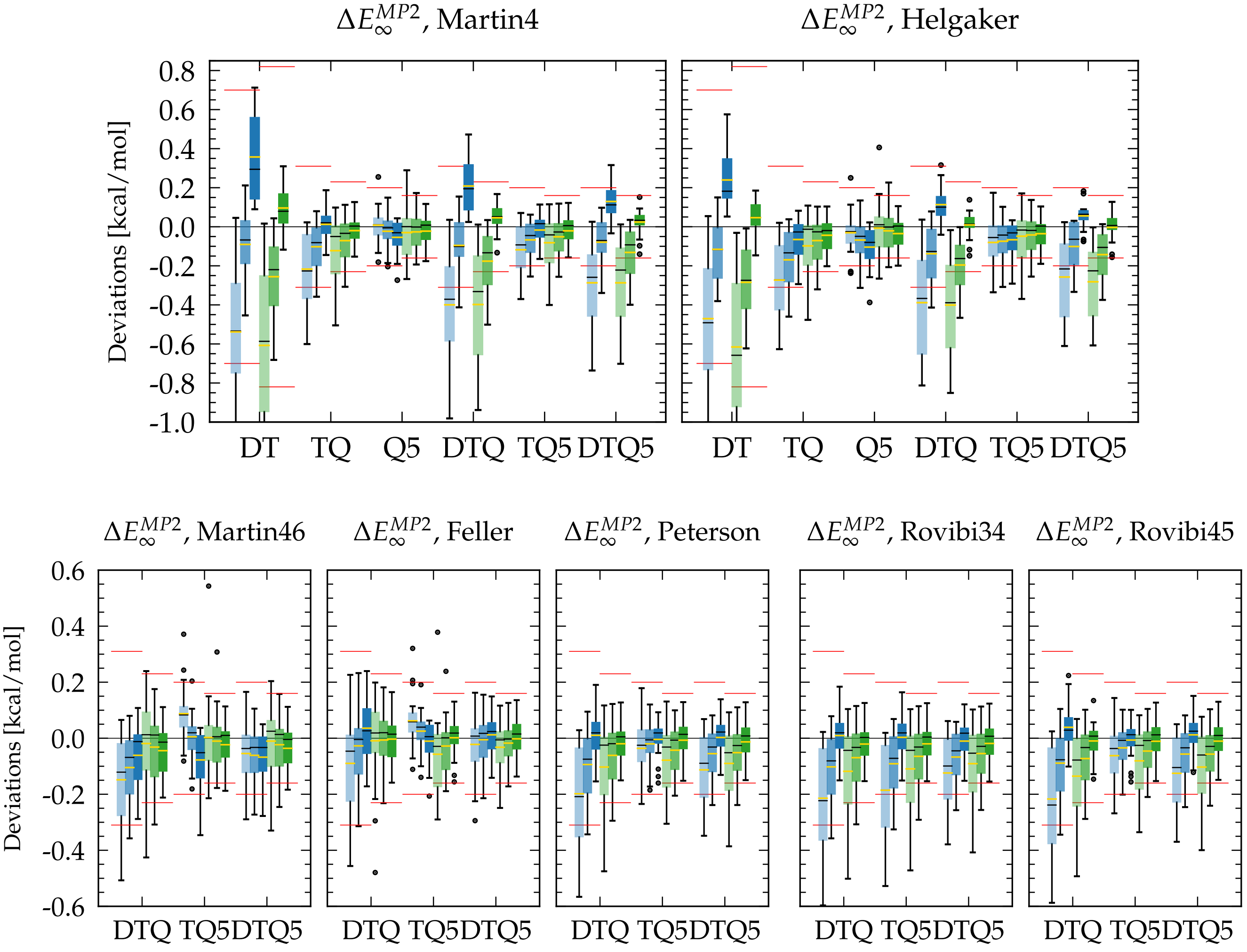

Figure S4: Box plots of the differences $\Delta E^{\mathrm{MP2}}_{\mathrm{GTO}} - \Delta E^{\mathrm{MP2}}_{\mathrm{PW}}$ between extrapolated GTO and PW MP2 interaction energies of the S22* test systems. Medians are shown as horizontal black lines and yellow lines stand for the mean signed deviation (MSD). The solid red lines correspond to the smallest maximum deviation obtained with plain T, Q or 5 zeta basis sets respectively, as reported in Table 2. The legend is given in Figure 2.

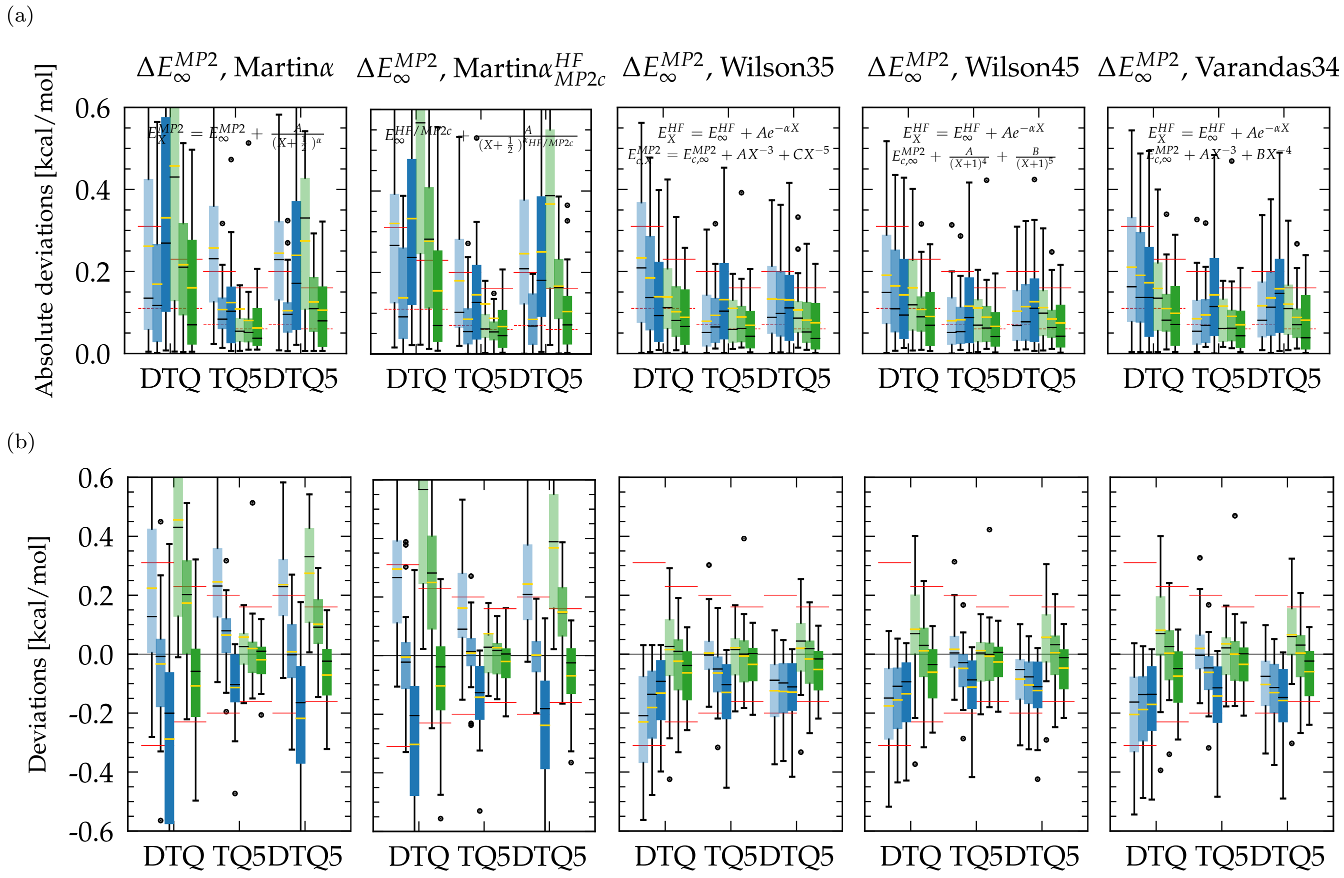

Figure S5: Box plots of the differences $\Delta E^{\text{MP2}}_{\text{GTO}} - \Delta E^{\text{MP2}}_{\text{PW}}$ between extrapolated GTO and PW MP2 interaction energies of the S22* test systems. If applicable, HF and MP2c contributions to the total MP2 energies have been extrapolated separately. Absolute differences are given in (a) while (b) reports signed values. Medians are shown as horizontal black lines and yellow lines stand respectively for the mean absolute error (MAE) in (a) and the mean signed deviation (MSD) in (b). The dashed(solid) red lines correspond to the smallest MAE(maximum deviation) obtained with plain Q or 5 zeta basis sets respectively, as reported in Table 2. The legend is given in Figure 2.

(a)

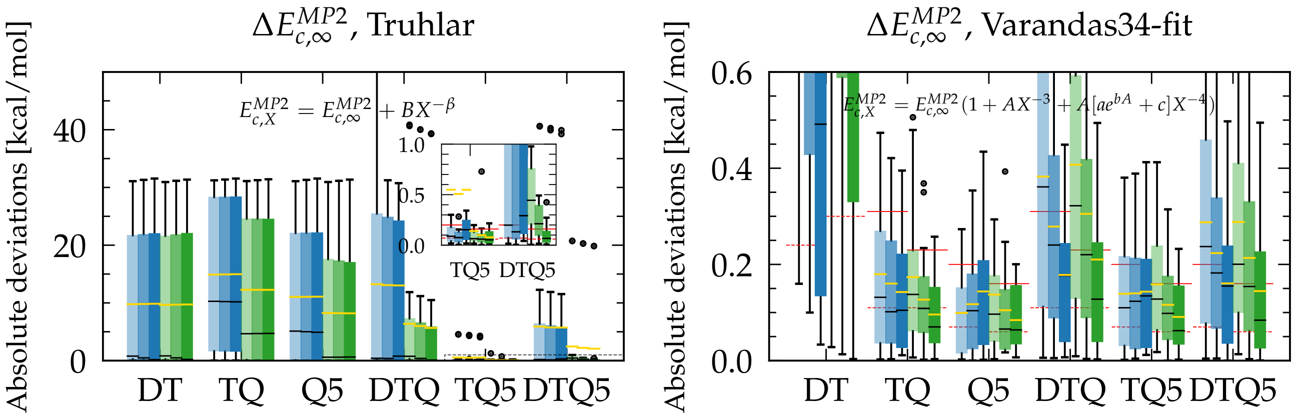

(b)

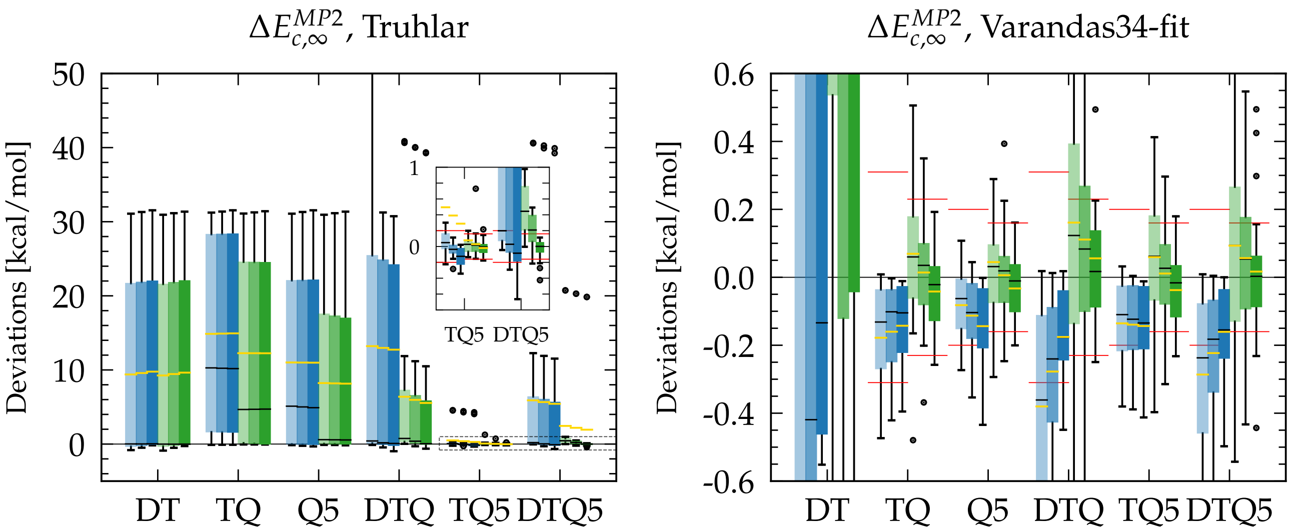

Figure S6: Box plots of the differences $\Delta E^{\mathrm{MP2}}_{c,\mathrm{GTO}} - \Delta E^{\mathrm{MP2}}_{c,\mathrm{PW}}$ between extrapolated GTO and PW MP2c interaction energies of the S22* test systems. Absolute differences are given in (a) while (b) reports signed values. Medians are shown as horizontal black lines and yellow lines stand respectively for the mean absolute error (MAE) in (a) and the mean signed deviation (MSD) in (b). The dashed(solid) red lines correspond to the smallest MAE(maximum deviation) obtained with plain T, Q or 5 zeta basis sets respectively, as reported in Table 2. The legend is given in Figure 2. Note that the small deviations due to the HF contribution would not counterbalance the results of MP2c.

(a)

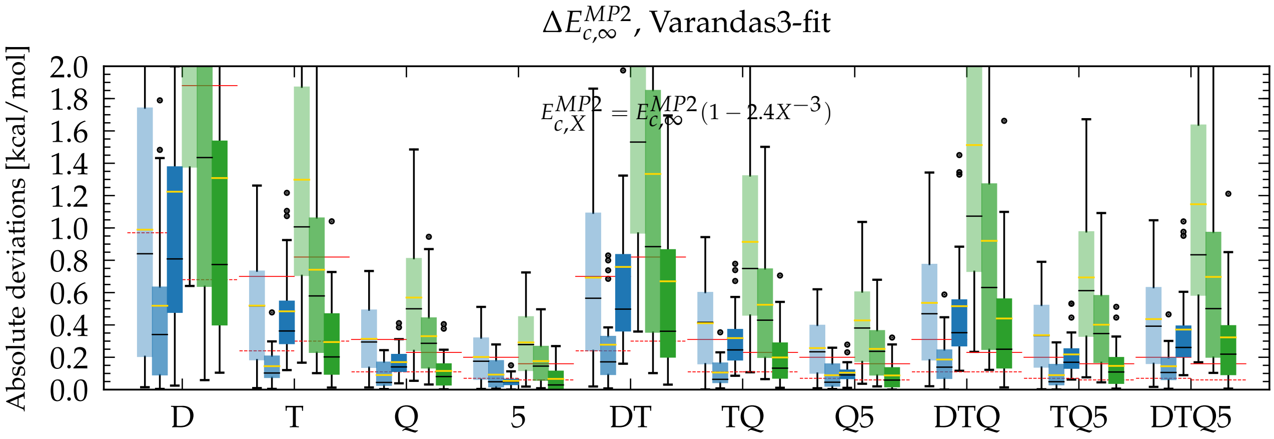

(b)

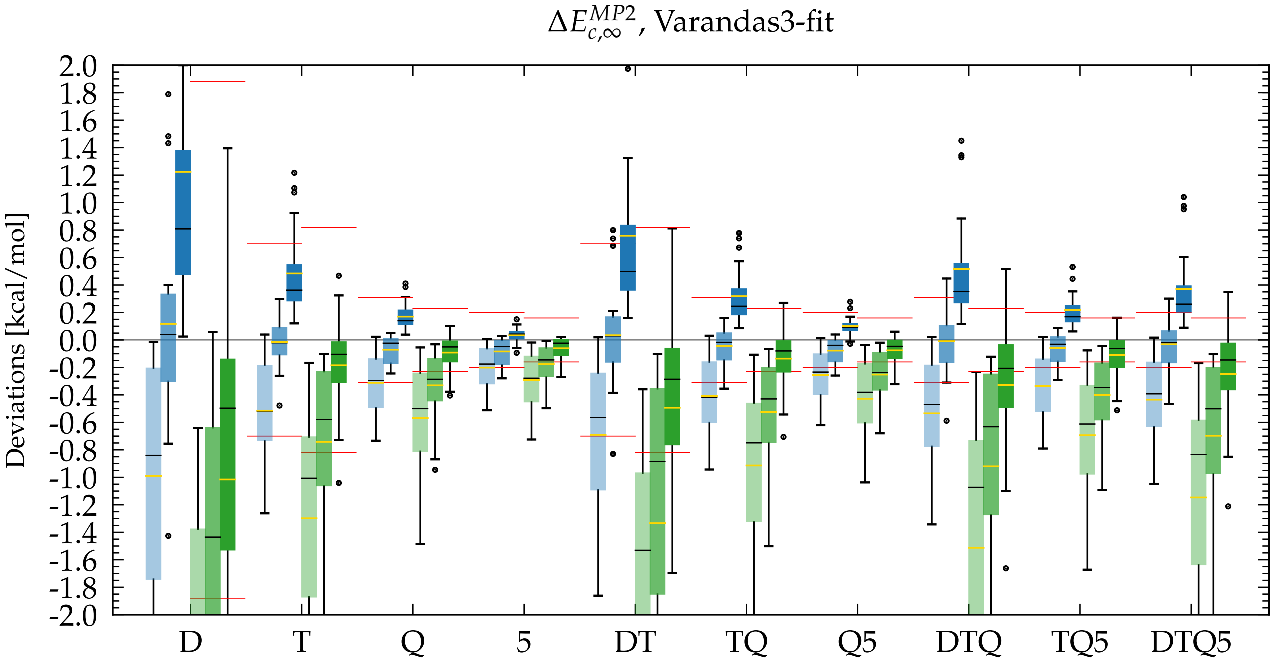

Figure S7: Box plots of the differences $\Delta E_{c,\mathrm{GTO}}^{\mathrm{MP2}} - \Delta E_{c,\mathrm{PW}}^{\mathrm{MP2}}$ between extrapolated GTO and PW MP2c interaction energies of the S22* test systems. Absolute differences are given in (a) while (b) reports signed values. Medians are shown as horizontal black lines and yellow lines stand respectively for the mean absolute error (MAE) in (a) and the mean signed deviation (MSD) in (b). The dashed(solid) red lines correspond to the smallest MAE(maximum deviation) obtained with plain D, T, Q or 5 zeta basis sets respectively, as reported in Table 2. The legend is given in Figure 2. Note that the small deviations due to the HF contribution would not counterbalance the results of MP2c.

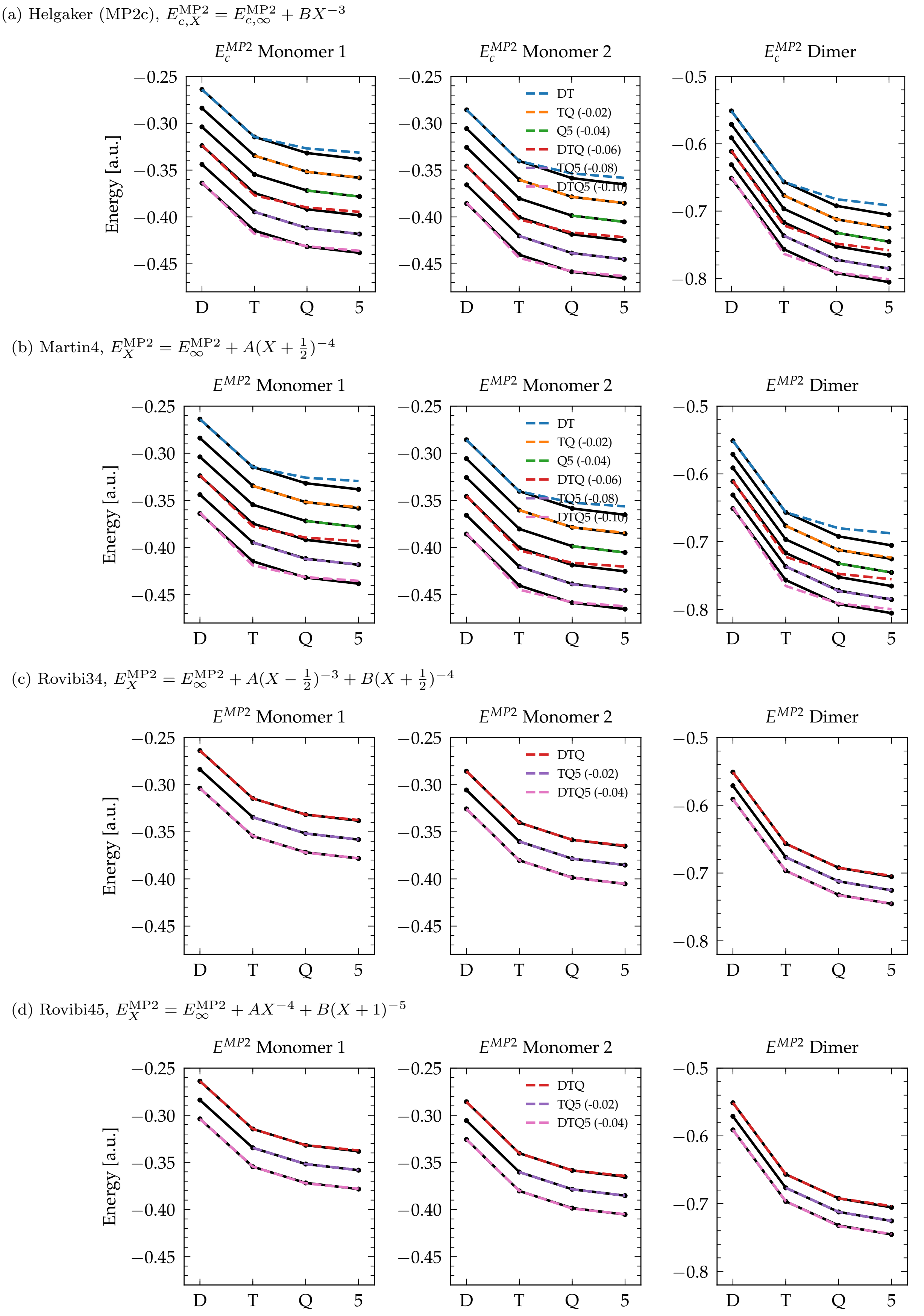

Figure S8: Examples of fitting curves on aug-cc-pVXZ/CP-corrected data points for the ethene-ethine complex. Including the D zeta point in the sequence for (a) *Helgaker* and (b) *Martin4* deteriorates the interpolation while these are better for (c) *Rovibi34* and (d) *Rovibi45*.

Table S3: Quality of interpolation per GTO extrapolation. The mean absolute error (MAE) and root-mean-square deviation (RMSD), as well as the coefficient of determination R^2 are calculated between fitting curves and (aug-)cc-pVXZ data points, always evaluated up to the 5 zeta values (like shown in Figure S8) to reflect the predictive power at larger Xs. Averages on all test systems are reported and include errors on both dimer and monomer energies that are fitted separately. MAE and RMSD are in [kcal/mol]. For simplicity and due to the small *Helgaker*/HF errors, *Helgaker*/MP2 results are based on the MP2c energies only.

Points	Set	Scheme	BSSE corr.	MAE	RMSD	R^2	Scheme	BSSE corr.	MAE	RMSD	R^2
HF - Helgaker vs Truhlar											
DTQ	non-aug	Helgaker	CP	0.058403	0.116806	0.999953	Truhlar	half-CP	0.353033	0.706064	0.999265
	aug	Helgaker	CP	0.099620	0.199236	0.999909	Truhlar	half-CP	0.374430	0.748858	0.998828
TQ5	non-aug	Helgaker	CP	0.000004	0.000005	0.999999	Truhlar	CP	0.000001	0.000001	0.999999
	aug	Helgaker	CP	0.000006	0.000007	0.999999	Truhlar	CP	0.000020	0.000022	0.999999
DTQ5	non-aug	Helgaker	CP	0.051213	0.062020	0.999986	Truhlar	CP	0.292207	0.352576	0.999814
	aug	Helgaker	CP	0.086686	0.104778	0.999974	Truhlar	CP	0.307017	0.370197	0.999712
MP2 - Helgaker vs Martin4											
		Best agreement with PWs according to Table 3					Best complementary Helgaker or Martin4				
DT	non-aug	Helgaker	half-CP	8.3797	12.0569	0.972987	Martin4	half-CP	10.5614	15.1795	0.976575
	aug	Helgaker	CP	6.6717	9.5909	0.977574	Martin4	CP	8.6849	12.4628	0.978886
TQ	non-aug	Martin4	CP	0.7062	1.2232	0.998475	Helgaker	CP	0.4706	0.8150	0.998973
	aug	Martin4	CP	0.4649	0.8052	0.999060	Helgaker	CP	0.3253	0.5635	0.999291
Q5	non-aug	Martin4	half-CP	$4 \cdot 10^{-9}$	$4 \cdot 10^{-9}$	1.000000	Helgaker	none	$6 \cdot 10^{-14}$	$7 \cdot 10^{-14}$	1.000000
	aug	Martin4	CP	$5 \cdot 10^{-9}$	$6 \cdot 10^{-9}$	1.000000	Helgaker	CP	$5 \cdot 10^{-14}$	$6 \cdot 10^{-14}$	1.000000
DTQ	non-aug	Helgaker	CP	5.9349	6.8382	0.991316	Martin4	half-CP	7.5922	8.7549	0.992187
	aug	Helgaker	CP	4.7317	5.4354	0.992792	Martin4	CP	6.2159	7.1282	0.993074
TQ5	non-aug	Martin4	CP	0.6558	0.7258	0.999463	Helgaker	CP	0.4303	0.4733	0.999654
	aug	Martin4	CP	0.4317	0.4778	0.999669	Helgaker	CP	0.2975	0.3272	0.999761
DTQ5	non-aug	Helgaker	CP	4.8106	5.8782	0.993582	Martin4	half-CP	6.1927	7.5687	0.994169
	aug	Helgaker	CP	3.8443	4.6838	0.994650	Martin4	CP	5.0918	6.1902	0.994785
MP2 - Rovibi34 vs Rovibi45											
DTQ	non-aug	Rovibi34	CP	0.2955	0.5910	0.999958	Rovibi45	CP	0.5313	1.0627	0.999872
	aug	Rovibi34	CP	0.1537	0.3075	0.999982	Rovibi45	CP	0.3601	0.7202	0.999919
TQ5	non-aug	Rovibi34	CP	$5 \cdot 10^{-10}$	$6 \cdot 10^{-10}$	1.000000	Rovibi45	CP	$1 \cdot 10^{-10}$	$2 \cdot 10^{-10}$	1.000000
	aug	Rovibi34	CP	$6 \cdot 10^{-10}$	$7 \cdot 10^{-10}$	1.000000	Rovibi45	CP	$3 \cdot 10^{-10}$	$4 \cdot 10^{-10}$	1.000000
DTQ5	non-aug	Rovibi34	CP	0.2699	0.3393	0.999986	Rovibi45	CP	0.4874	0.6121	0.999958
	aug	Rovibi34	CP	0.1404	0.1765	0.999994	Rovibi45	CP	0.3303	0.4149	0.999973
MP2 - Peterson vs Feller											
DTQ	non-aug	Peterson	CP	0.0691	0.1381	0.999996	Feller	CP	0.4250	0.8501	0.999887
	aug	Peterson	CP	0.1317	0.2634	0.999989	Feller	CP	0.3234	0.6468	0.999914
TQ5	non-aug	Peterson	CP	$1 \cdot 10^{-7}$	$2 \cdot 10^{-7}$	1.000000	Feller	CP	$9 \cdot 10^{-7}$	$1 \cdot 10^{-6}$	1.000000
	aug	Peterson	CP	$9 \cdot 10^{-9}$	$1 \cdot 10^{-8}$	1.000000	Feller	CP	$4 \cdot 10^{-6}$	$4 \cdot 10^{-6}$	1.000000
DTQ5	non-aug	Peterson	CP	0.0618	0.0767	0.999999	Feller	CP	0.3460	0.4083	0.999973
	aug	Peterson	CP	0.1178	0.1463	0.999997	Feller	CP	0.2648	0.3130	0.999979

(a) Non-extrapolated

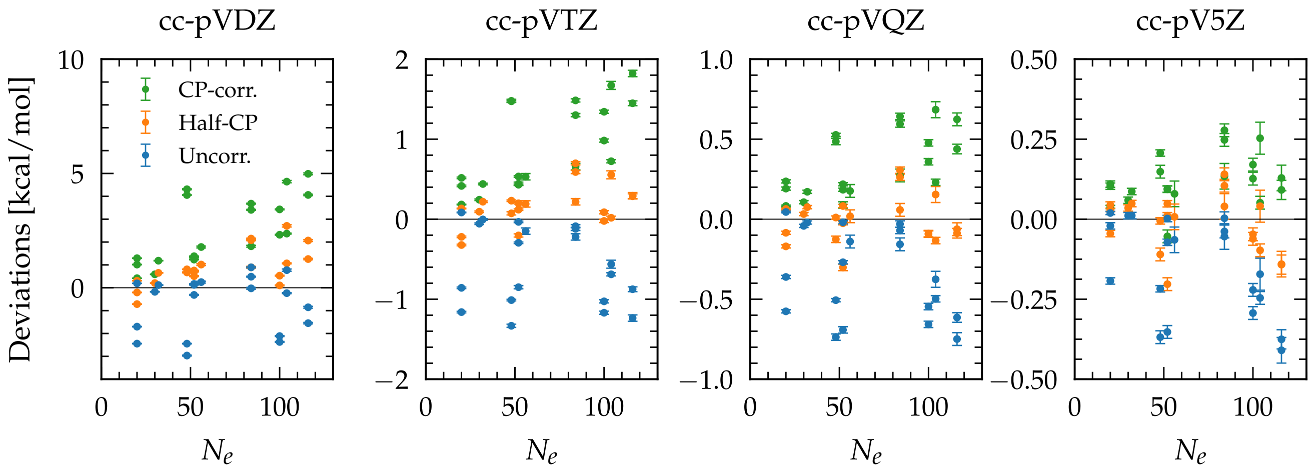

(b) Extrapolated, closest to PWs in the CBS limit

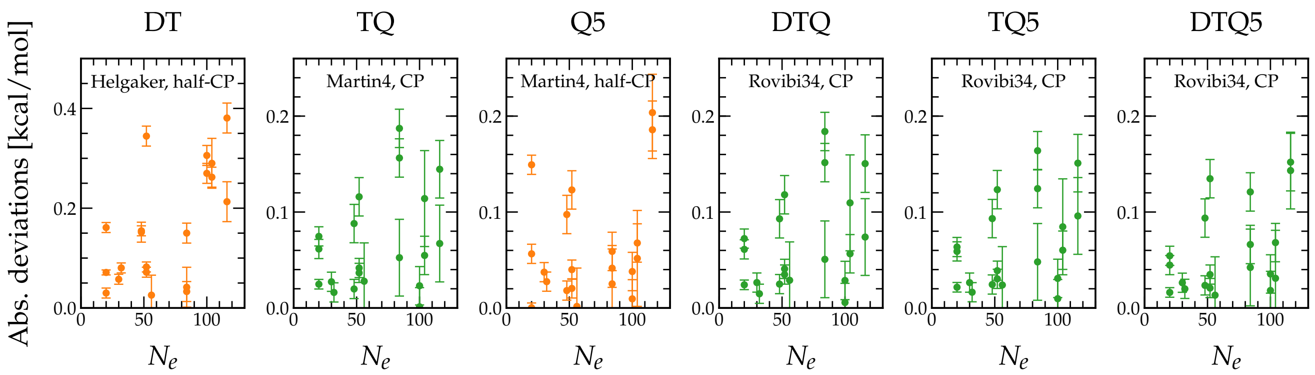

Figure S9: Deviations between the cc-pVXZ and PW MP2 interaction energies as a function of the number of electrons N_e in the dimer system. (a) for plain basis sets, (b) for energies extrapolated to the CBS limit with best schemes of Tables 3 and 4.

(a) cc-pVXZ, Martin4

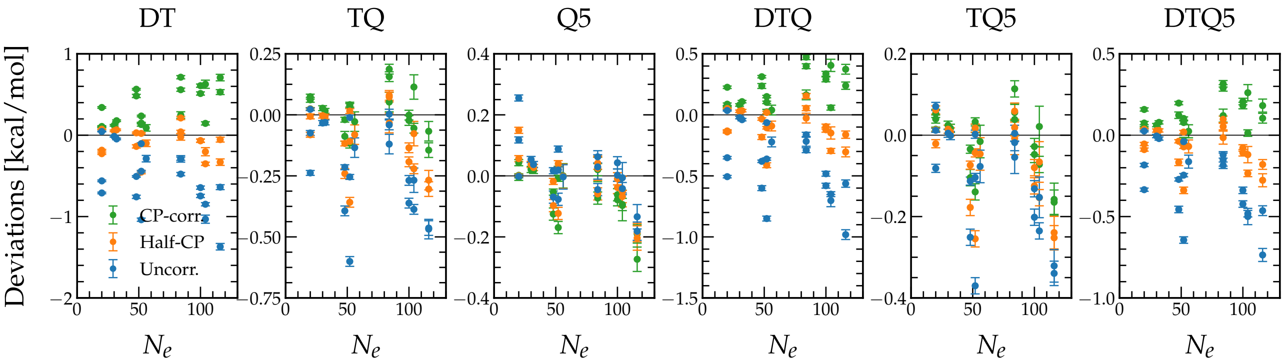

(b) cc-pVXZ, Helgaker

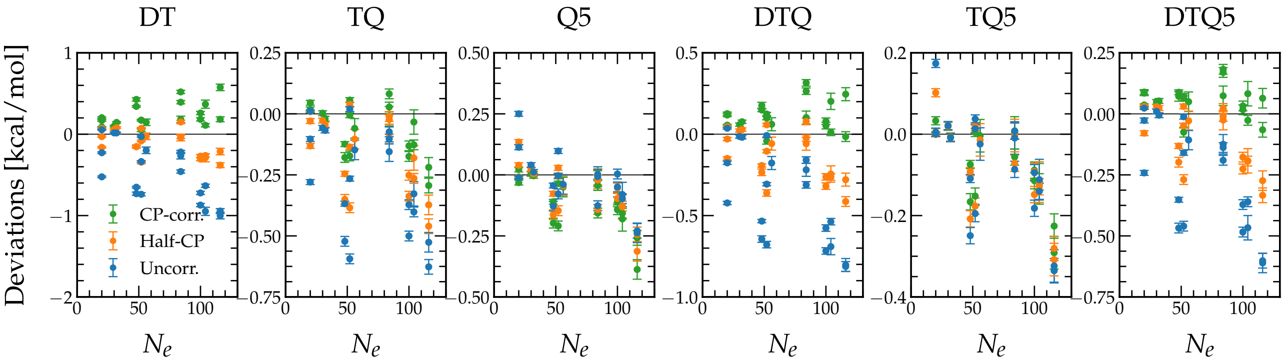

(c) cc-pVXZ, Peterson

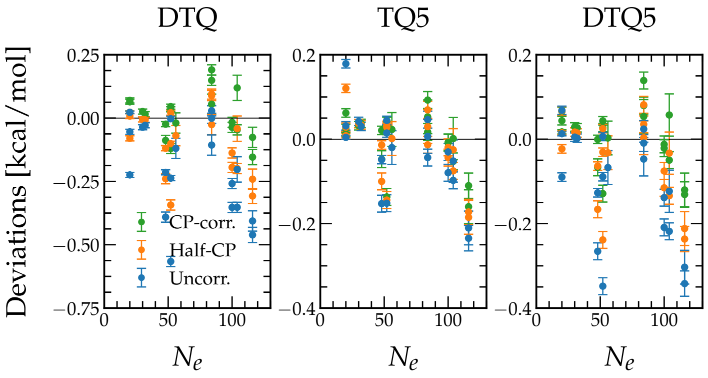

(d) cc-pVXZ, Rovibi34

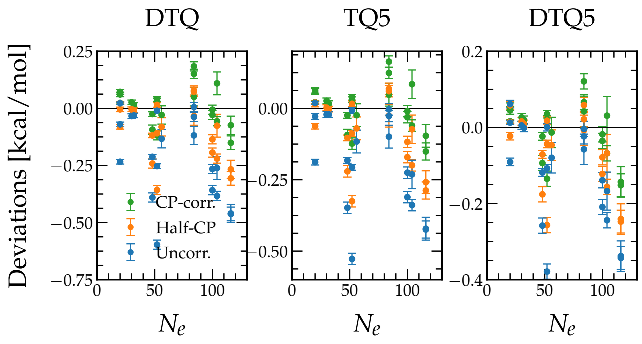

(e) cc-pVXZ, Rovibi45

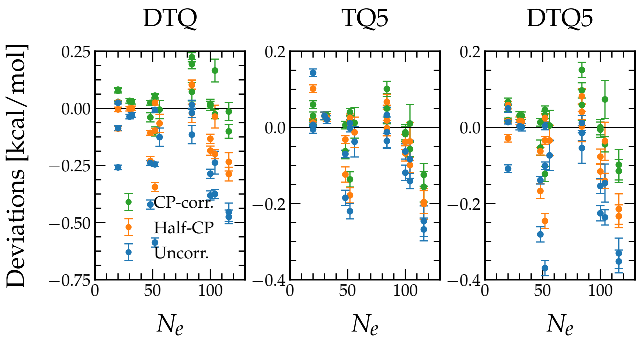

Figure S10: Deviations between the extrapolated cc-pVXZ and PW MP2 interaction energies as a function of the number of electrons N_e in the dimer system. Extrapolated to the CBS limit with (a) Martin4, (b) Helgaker, (c) Peterson, (d) Rovibi34, (e) Rovibi45. For all schemes, the deviations (in absolute value) tend to increase with N_e.

(a) aug-cc-pVXZ, Martin4

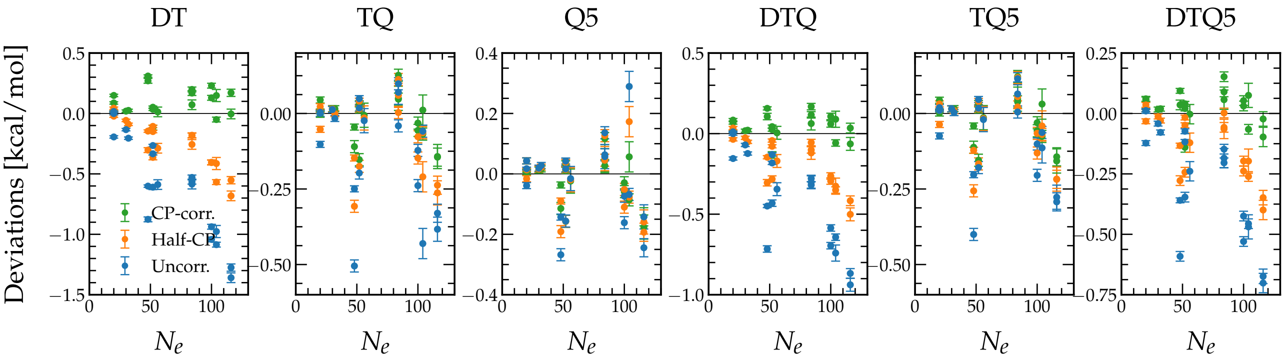

(b) aug-cc-pVXZ, Helgaker

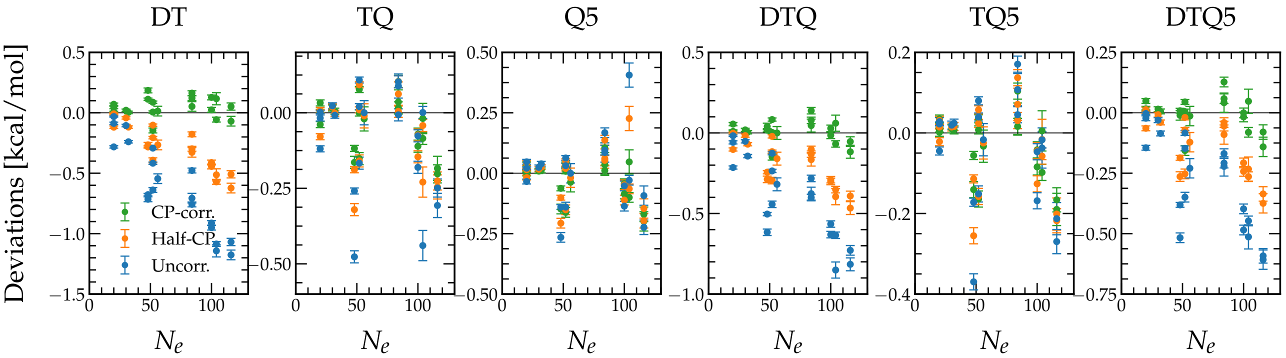

(c) aug-cc-pVXZ, Peterson

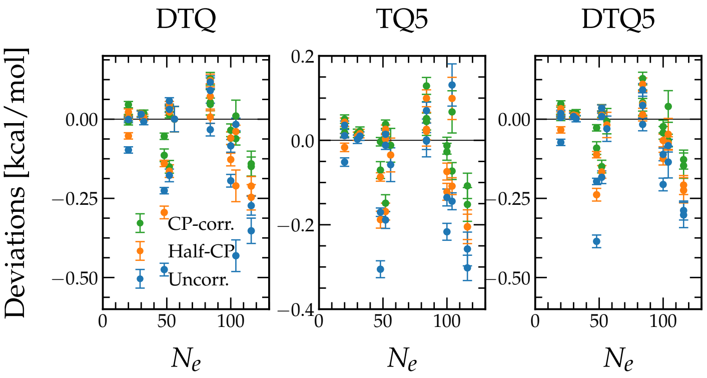

(d) aug-cc-pVXZ, Rovibi34

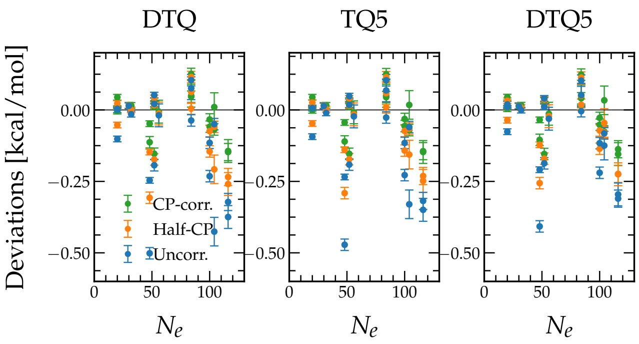

(e) aug-cc-pVXZ, Rovibi45

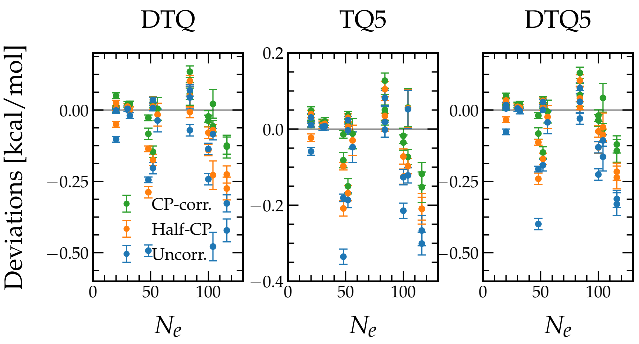

Figure S11: Deviations between the extrapolated aug-cc-pVXZ and PW MP2 interaction energies as a function of the number of electrons N_e in the dimer system. Extrapolated to the CBS limit with (a) Martin4, (b) Helgaker, (c) Peterson, (d) Rovibi34, (e) Rovibi45. For all schemes, the deviations (in absolute value) tend to increase with N_e.
